# Characterising covalent warhead reactivity

**DOI:** 10.1016/j.bmc.2019.04.002

**Published:** 2019-05-15

**Authors:** James S. Martin, Claire J. MacKenzie, Daniel Fletcher, Ian H. Gilbert

**Affiliations:** Drug Discovery Unit, Wellcome Centre for Anti-Infectives Research, Division of Biological Chemistry and Drug Discovery, University of Dundee, Dundee DD1 5EH, UK

**Keywords:** Covalent drug, Warhead, Reactivity

## Abstract

Many drugs currently used are covalent inhibitors and irreversibly inhibit their targets. Most of these were discovered through serendipity. Covalent inhibitions can have many advantages from a pharmacokinetic perspective. However, until recently most organisations have shied away from covalent compound design due to fears of non-specific inhibition of off-target proteins leading to toxicity risks. However, there has been a renewed interest in covalent modifiers as potential drugs, as it possible to get highly selective compounds. It is therefore important to know how reactive a warhead is and to be able to select the least reactive warhead possible to avoid toxicity. A robust NMR based assay was developed and used to measure the reactivity of a variety of covalent warheads against serine and cysteine – the two most common targets for covalent drugs. A selection of these warheads also had their reactivity measured against threonine, tyrosine, lysine, histidine and arginine to better understand our ability to target non-traditional residues. The reactivity was also measured at various pHs to assess what effect the environment in the active site would have on these reactions. The reactivity of a covalent modifier was found to be very dependent on the amino acid residue.

## Graphical abstract

**Figure:**
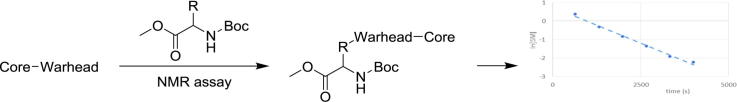

## 1 Introduction

**Fig. 1:**
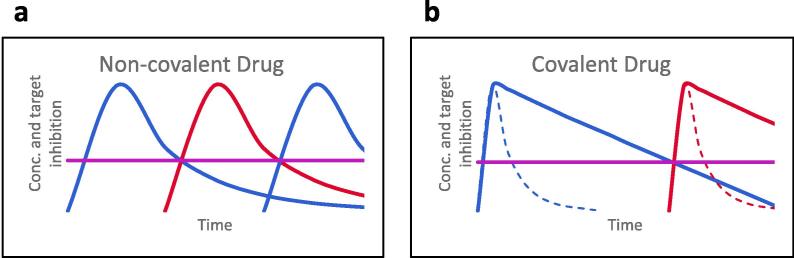
(a) With a non-covalent drug (left) the concentration must usually be kept above the minimum efficacious dose (purple line) to have an effect. The concentration and target inhibition are directly related. (b) With a covalent drug once the covalent bond has formed it is not necessary to maintain the free drug (dashed line) in the body at high concentrations as the drug is irreversibly bound to the target (solid line) until the target is degraded. In this case the drug concentration is not directly related to target inhibition. (For interpretation of the references to colour in this figure legend, the reader is referred to the web version of this article.)

Covalent drugs act to inhibit enzymes irreversibly through formation of a covalent bond, typically to the reactive side chain of an amino acid in the enzyme active site.^1^ Until recently, many organisations have avoided covalent modifiers in drug discovery, due to concerns of non-specific modification of other proteins giving rise to toxicity. However, there is now renewed interest in covalent modifiers as drugs. Indeed many current drugs are covalent inhibitors, but most of these have not been designed, but discovered by serendipity. Covalent drugs include for example β-lactam antibiotics,^2^ aspirin,^3^, ^4^ clopidogrel,^4^ osimertinib,^5^ and omeprazole.^6^ Covalent drugs can provide advantages including an increased residency time at the molecular target compared to “traditional reversible” inhibitors,^7^ which can lead to a dosing regimen, where the concentration of the free drug does not need to be constantly maintained above the efficacious dose as shown in Fig. 1.

By careful design of the molecule, excellent selectivity can be obtained. When designing a covalent drug it may be useful to consider binding as occurring in two distinct stages to avoid toxicity.^8^ First the drug has to bind to the target via non-covalent bonds and for this it depends on the overall structure of the binding site as is the case with a traditional reversible drug. Then a covalent drug has to form a covalent bond to a specific nucleophilic residue in the target. The non-covalent binding has to be optimised through design of the overall compound structure. However, the second part is optimised by careful selection of the covalent warhead to make sure that it has appropriate reactivity and orientation within the active site. The warhead should have sufficient reactivity to form the covalent bond to the residue in the active site, when held in the correct orientation by the recognition motif, but insufficient reactivity to non-specifically react with residues in other proteins.

**Fig. 2:**
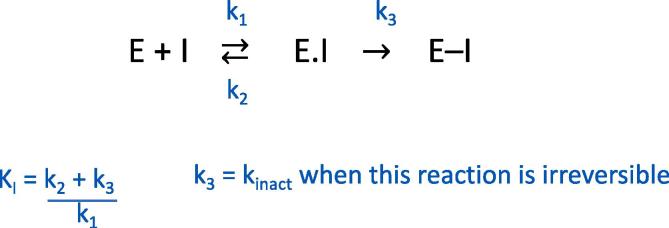
The kinetic models used to explain covalent inhibition.

Kinetic models have been introduced to explain covalent inhibition as shown in Fig 2.^9^, ^10^ In the first step, the enzyme and inhibitor form a reversible complex (E.I). There is then a second step in which the covalent modifier forms a covalent bond with the enzyme. In this model the rate at which covalent bond formation occurs is defined as k_inact_/K_I_, where: k_inact_ is the maximum rate at which the reversible complex forms the covalent bond (k_3_); and K_I_ is defined as (k_2_ + k_3_)/k_1_ and is the concentration of inhibitor which gives half the maximum rate of covalent bond formation (E–I).

However, other factors may affect the process. For example, it is possible in some cases that there is a direct reaction between the covalent inhibitor and the protein without a prior molecular recognition event. In other cases the protein may have a substantial role in covalent inhibitor bond formation.^11^

The main concern with covalent modifiers is their ability to bind irreversibly to off target proteins and result in toxicological effects such as immune responses.^12^ To better understand this risk, and have a logical approach to reduce these effects, it is important to have an understanding of the reactivity of typical covalent warheads. Previous assays to measure the reactivity of covalent warheads have either focused on cysteine^13^, ^14^, ^15^ (for example using fluorogenic probes)^14^ or glutathione (using a mixture of computational [quantum mechanical] and experimental [LCMS] approaches)^12^ or focused on a single type of covalent warhead.^16^ By looking at a range of covalent warheads and a range of amino acids, a better understanding of the relative reactivity can be obtained. To this end a NMR based assay was designed which allows the rate of reaction of any amino acid with any covalent warhead of interest to be measured. The reactivities of a selection of common covalent warheads were measured against cysteine and serine as these are the most commonly targeted amino acids. A selection of other potentially reactive amino acids was also investigated.

It should be noted that the reactivity of the covalent warheads measured here was carried out in solution. However in the context of an enzyme, molecular recognition events will constrain the warhead to a limited set of orientations within the active site. The orientation of the warhead will affect the rate at which it reacts with the protein. In an ideal scenario, the warhead should be constrained in orientation to give a favourable trajectory for reacting with the appropriate residue, which should result in the covalent bond formation reaction occurring faster and help reduce off target effects.^16^ The measurements reported here cannot replicate the molecular recognition events, but are useful for comparing the reactivity of different covalent warheads to a variety of nucleophiles, which is important in selecting potential warheads.

## 2 Results

### 2.1 Kinetics assay

**Fig. 3:**
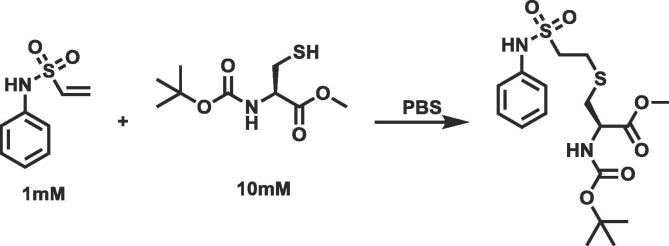
An example kinetic assay.

An NMR assay which can track the amount of the covalent warhead in solution with an amino acid was designed. A typical assay setup is shown in Fig. 3.

The amino acid is used at 10x the concentration of the warhead to give the reaction pseudo first order kinetics, as the concentration of the amino acid remains in large excess of the warhead throughout the reaction and therefore can be considered to be constant. To ensure that the covalent warhead was reacting with the sulfur of the cysteine or the hydroxyl of the serine the amine group was protected with a BOC group and the carboxylic acid was protected as a methyl ester.

**Fig. 4:**
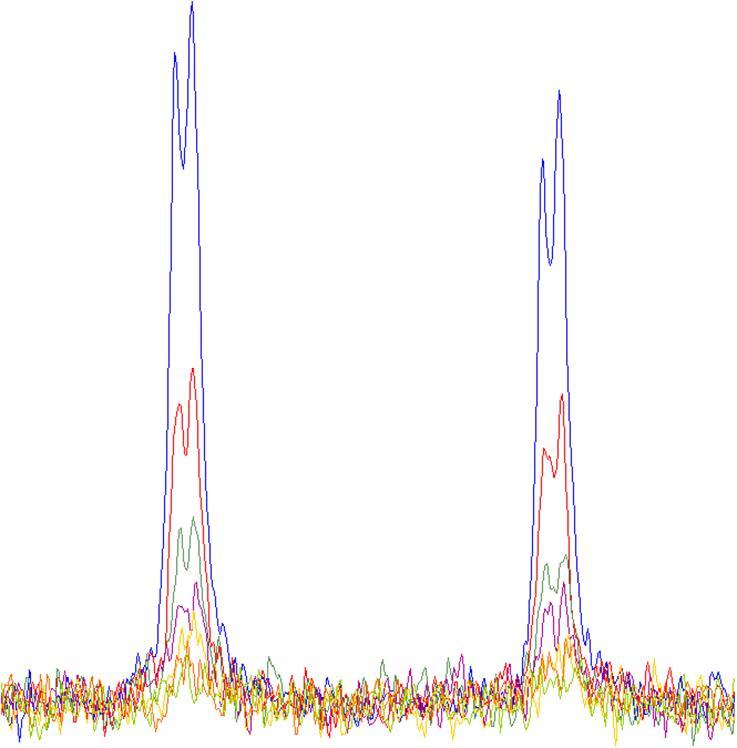
Example NMR spectra for the reaction shown in Fig. 3 looking at one of the vinyl protons. Over time the amount of covalent warhead is reduced and this is observed as the area under these peaks decreasing.

As the reaction proceeds the amount of the covalent warhead is reduced and this can be measured as the decrease in the integral of the NMR peaks corresponding to the warhead as seen in Fig. 4. The peaks selected for monitoring by NMR are those where the chemical shift will change substantially between the substrate and the product. Typically these were peaks corresponding to atoms associated with the warhead; however, peaks in the attached phenyl ring can also be used when required. A suitable relaxation delay was included in the experimental design, to minimise saturation effects. All this ensures that the experiments are quantitative. The rates of reaction were relatively slow for most reactions, meaning the setup, data acquisition and mixing times were not significant and would not affect the rate determination, which is determined by the gradient of the line obtained.

**Fig. 5:**
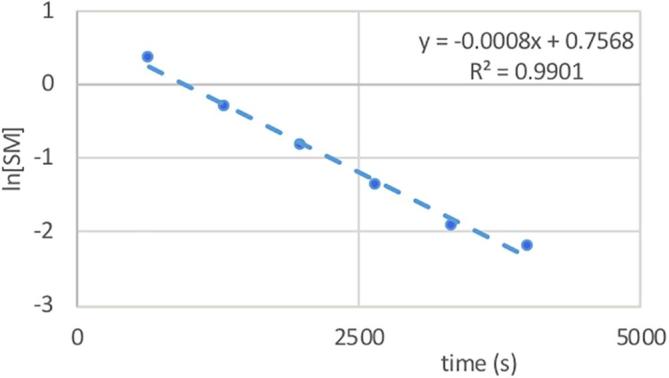
Results from the example kinetic assay.

By then plotting the natural log of the integral of these peaks against time (Fig. 5) a straight line is achieved where the gradient corresponds to the rate of the reaction. By doing this for each peak corresponding to the covalent warhead in the NMR spectra an accurate measure of the reactivity can be achieved.

### 2.2 Results with cysteine and serine

**Fig. 6:**
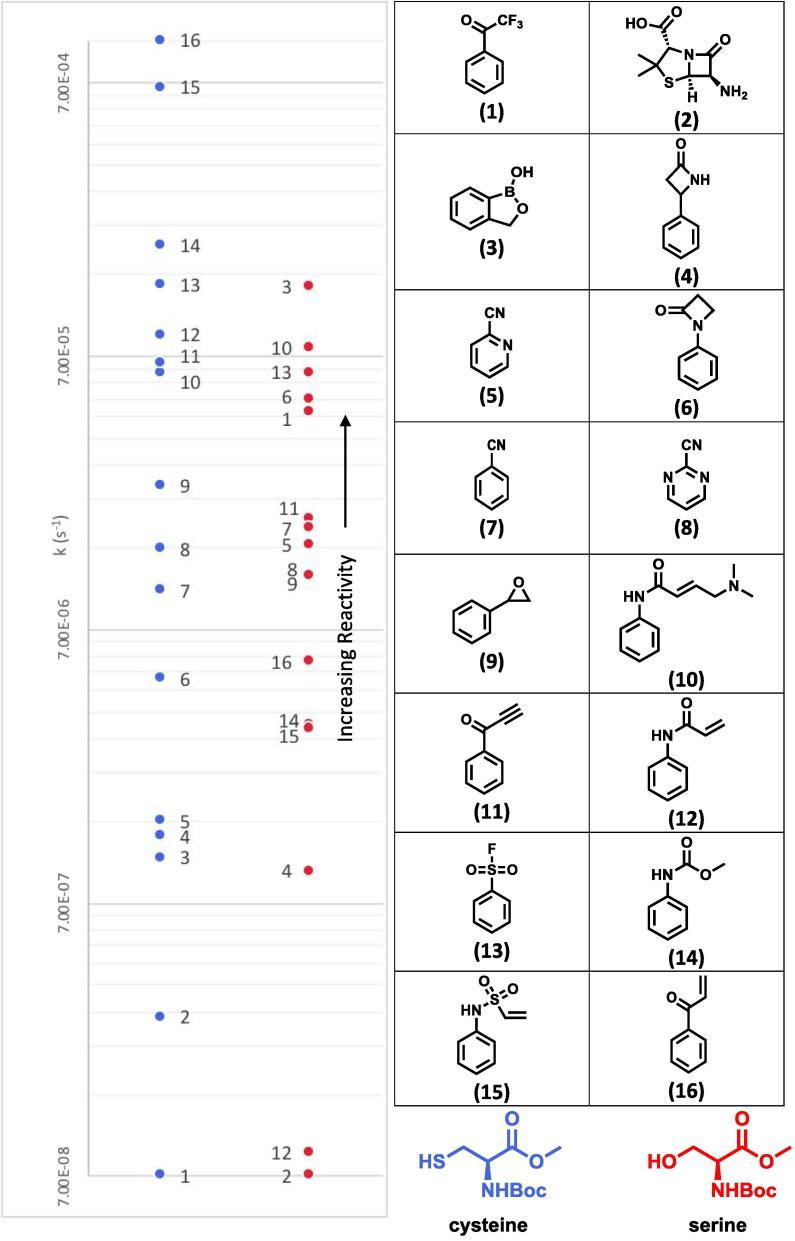
The results of measuring the reactivity of covalent warheads against cysteine (blue dots) and serine (red dots). Compound numbers correspond to their number in the graph and are numbered sequentially based on their reactivity with cysteine (1 = least reactive, 16 = most reactive). (For interpretation of the references to colour in this figure legend, the reader is referred to the web version of this article.)

The reactivity of a range of covalent warheads measured against cysteine and serine are presented in Fig. 6. The blue points correspond to the rate constant for the reaction with cysteine and the red points the rate constant for the reaction with serine. Each point is numbered according to the compound in the accompany table to Fig. 6.

**Fig. 7:**
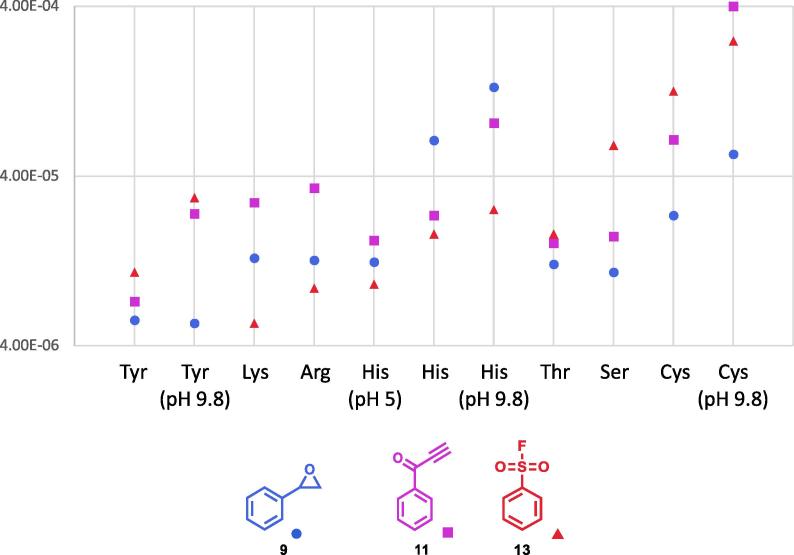
The measured rate constants for selected covalent warheads reacting with potential target amino acids.

The reactivity of a selection of these warheads against other amino acids which could also be targeted are presented in Fig. 7. All assays were initially performed at physiological pH (pH = 7.4). However, many residues in active sites have perturbed pKa values which change the reactivity to electrophiles and their protonation state. Amino acids with a pKa where it was conceivable that the protonation state could be substantially changed were investigated at various pHs. Histidine (pKa = 6) was investigated at pH 5 and pH 9.8 and cysteine (pKa = 8) and tyrosine (pKa = 10) were both also investigated at pH 9.8. The results are also shown in Fig. 7. To ensure the assay is reproducible the rate of reaction between cysteine and both the acrylamide (**15**) and the vinyl sulfonamide (**16**) was measured three times and an average taken. In each case the average was found to be within 7% of the measured rates suggesting that the assay is extremely reproducible (Table 1). Due to the time required for each experiment and the reproducibility of the assay each experiment was performed once for other examples.

**Table 1:** Activity of covalent warheads with cysteine; data in triplicate.

Covalent Warhead	
	k = 8.38 × 10^−5^ s^−1^
	t_1/2_ = 2.3 ± 0.1 h
	k = 6.73 × 10^−4^ s^−1^
	t_1/2_ = 17.2 ± 1.2 min

During this study we have looked at both potentially “reversible” and “irreversible” covalent modifiers. A “reversible” covalent inhibitor would be for example the trifluoroketone (**1**), the oxaborole (**3**) and the nitriles (**5**, **7**, **8**, **9**), where a reversible reaction could potentially regenerate the inhibitor. The Michael acceptors (**10**, **11**, **12**, **15**, **16**) are likely also reversible, albeit the rate of the reverse reaction is likely to be very slow and in many cases essentially irreversible. The irreversible warheads are the β-lactams (**2**, **4**, **6**), where hydrolysis of the inhibitor from the amino acid residue could potentially happen, but would produce a modified and unreactive version of the inhibitor.

It is instructive to compare reactions with cysteine and serine. There is a very different order in reactivity of the different warheads with the two residues. This can be largely explained as serine is a much “harder” nucleophile than cysteine and tends to react faster with the “harder” electrophiles (for example the benzoxaborole (**3**) and the sulfonyl fluoride (**13**) are “harder” electrophiles). In contrast, the Michael acceptors **12**, **15** and **16** are softer electrophiles and react more rapidly with the “softer” cysteine. The cysteine reacts with these Michael acceptors at least 2 orders of magnitude more rapidly than serine does. The reactions of cysteine with **15** and **16** are significantly faster than any of the other reactions that we investigated. Compound **16** was found to react so quickly with cysteine that a rate constant could not be measured for this reaction. The “hard” or “soft” natures of the electrophiles was suitable for prediction of the relative reactivity between cysteine and serine in the majority of cases, but in some cases, particularly in cases where the electrophile was of moderate “hardness” or “softness” the relative reactivity was more difficult to predict and other factors may be important.

The acrylamide warhead (**12**) is currently under investigation in a range of ongoing covalent modifier drug discovery projects,^17^ this was found to react 3 orders of magnitude faster with cysteine than with serine suggesting why it is of such interest. However, this is not observed with the closely related compound **10** which was found to have very similar reactivity between cysteine and serine. This may be because the nitrogen in compound **10** alters the reactivity of the double bond or it may be that the nitrogen is able to act as a general base and deprotonate the serine to make it more reactive. Compound **11** also had similar reactivity with both cysteine and serine.

The nitriles (**5**, **7**, **8**) appear to react similarly with both cysteine and serine, but have relatively slow rates. The sulfonyl fluoride (**13**) was quite reactive to both cysteine and serine. The carbamate (**14**) was significantly more reactive to cysteine than serine.

Compound **2** (a penicillin) was found to react with serine too slowly for a rate to be measured. This is interesting because penicillins, like compound **2**, are known to react with a serine residue *in vivo* to have an effect. This shows how important the non-covalent binding and orientation of the warhead in active site plays in the ability of covalent drugs to form a covalent bond to their target. The β-lactams **4** and **6** were more reactive than the penicillin with both cysteine and serine. Interestingly compound **6** was moderately reactive with serine. This may be due to the nitrogen lone pair being less available due to delocalisation into the phenyl ring, increasing the reactivity of the carbonyl.

A wide range of different covalent warheads, with different reactivities, are found in clinically used drugs. This shows that the reactivity of the warheads depends on a number of factors, including the amino acid residues found in the target and the recognition motif that gives rise to the initial non-covalent binding interaction and selectivity with the target.

### 2.3 Other amino acids

Tyrosine, arginine, lysine, threonine and histidine were identified as amino acids that might also be targetable using covalent inhibitors. To investigate this, three covalent warheads which displayed the overall trend of being more reactive against cysteine than serine in Fig. 6 were selected and their reactivity measured against these amino acids. It was found that all amino acids were targetable to various degrees with histidine being as reactive as serine while tyrosine was found to be far less reactive. It is likely that other covalent warheads could be identified that are better at targeting these amino acids.

Amino acids where it is conceivable that there could be significantly different protonation states in proteins were also investigated at other pHs. Histidine (pka = 6) was investigated at pH 5 and pH 9.8. Tyrosine (pKa = 10) and cysteine (pKa = 8) were also investigated at pH 9.8. As was expected where the amino acids were more deprotonated, and therefore more reactive, the reactions proceeded faster.

### 2.4 General comments

Ultimately, the ability to target a particular amino acid will depend on both the warhead being used and on the environment in which the residue exists.

Overall these results provide an indication of how a drug containing a covalent warhead may be tuned to a particular project. If a covalent compound is too reactive and is found to have toxic side effects a less reactive warhead can be selected. Conversely, if the covalent warhead is not reactive enough to form a bond to the target then a more reactive warhead can be selected. Also we have derived information in understanding the differences between the different amino acid residues. The relative orientation of the warhead to the nucleophile in the binding site will also be an important factor in the success of a covalent drug, which has not been assessed here. The advantages of this approach is that it is simple to carry out and gives an indication of the relative reactivity of different warheads with different amino acid side chains. However, it does not take account of the molecular recognition events within an enzyme active site.

### 2.5 Synthesis

**Scheme 1:**
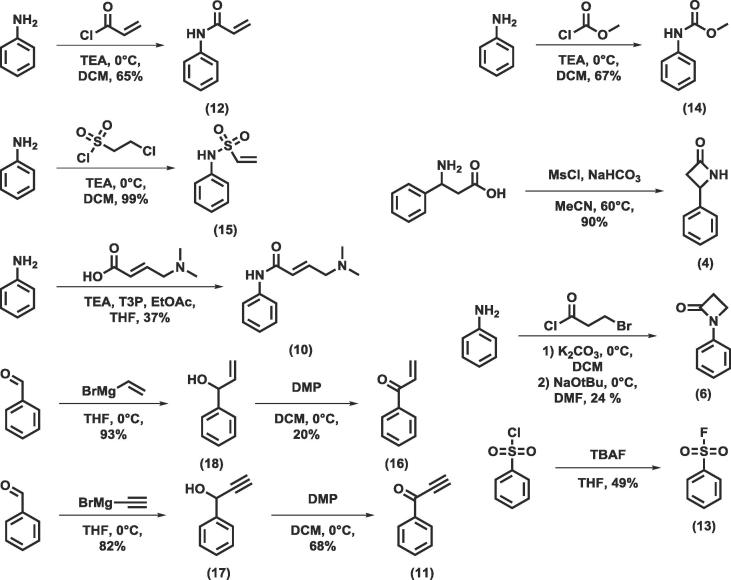
Synthesis of the covalent warheads of interest.

Some of the covalent warheads of interest were commercially available and these were purchased. Those which were not available were synthesised as shown in Scheme 1. The acrylamide **(12)** was synthesised from aniline using acryloyl chloride and triethylamine in dichloromethane at 0 °C. The methyl carbamate **(14)** was obtained under the same conditions using methyl chloroformate, as was the vinyl sulfonamide **(15**) using the sulfonyl chloride. The substituted acrylamide **(10)** was synthesised from aniline and the appropriate carboxylic acid using propylphosphonic anhydride as the coupling reagent in tetrahydrofuran. The 4-β-lactam **(4)** was synthesised from β-phenylalanine using mesyl chloride and sodium bicarbonate in acetonitrile at 60 °C. The 1-β-lactam **(6)** was obtained from aniline using 3-bromopropionyl chloride and potassium carbonate in dichloromethane at 0 °C to give the amide which was then cyclised using sodium *tert*-butoxide in dimethylformamide at 0 °C. The ketones **(11, 16)** were synthesised from benzaldehyde using the appropriate Grignard reagent in tetrahydrofuran at 0 °C. These were then oxidised to the ketone using Dess-Martin periodinane. Finally, the sulfonyl fluoride **(13)** was obtained by treating the sulfonyl chloride with TBAF in tetrahydrofuran.

**Scheme 2:**
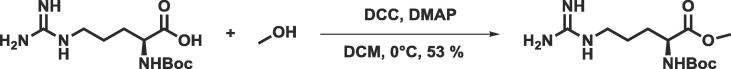
Synthesis of Boc-Arg-OMe.

The amino acids of interest were commercially available with the exception of Boc-Arg-OMe. This was synthesised as shown in Scheme 2 using DCC.

## 3 Experimental

### 3.1 General methods

Chemicals and solvents were purchased from commercial sources and were used without any further purification. Air and water sensitive reactions were carried out under an inert nitrogen atmosphere in oven dried glassware. Analytical thin-layer chromatography (TLC) was performed on precoated TLC plates (layer 0.20 mm silica gel 60 with fluorescent indicator UV254, from Merck). Developed plates were air-dried and analysed under a UV lamp (UV254/365 nm) and by staining with permanganate or ninhydrin. Flash column chromatography was performed on prepacked silica gel cartridges (230–400 mesh, 40–63 μm, from SiliCycle) using a Teledyne ISCO Combiflash Rf or Combiflash Rf 200i.^1^H (400 MHz or 500 MHz), ^13^C (100 MHz or 125 MHz), and 2D NMR spectra were recorded in CDCl_3_, MeOD or DMSO‑*d*_6_ using a Bruker Avance spectrometer. Proton chemical shifts are reported in ppm relative to the residual chloroform peak (δ = 7.26 ppm), methanol peak (δ = 3.31 ppm) or DMSO peak (δ = 2.50 ppm). Multiplicities are given as s (singlet), d (doublet), t (triplet), q (quartet), qui (quintet), m (multiplet), brs (broad singlet), dd (doublet of doublets), td (triplet of doublets), dt (doublet of triplets) or as a combination of these. Coupling constants (*J*) are quoted to the nearest 0.1 Hz. ^13^C chemical shifts are reported in ppm relative to the residual chloroform peak (δ = 77.16 ppm), methanol peak (δ = 49.00 ppm) or DMSO peak (δ = 39.51 ppm). Assignment of proton and carbon signals was achieved using COSY, HSQC and HMBC experiments. LCMS analysis was performed with either an Agilent HPLC 1100 series connected to a Bruker Daltonics MicrOTOF or an Agilent Technologies 1200 series HPLC connected to an Agilent Technologies 6130 quadrupole spectrometer, where both instruments were connected to an Agilent diode array detector. High resolution electrospray measurements were performed on a Bruker Daltonics MicrOTOF mass spectrometer. Preparative HPLC was performed on a Gilson HPLC (321 pump, 819 injection module, 215 liquid handler/injector) connected to a Gilson 155 UV/vis detector using Waters XBridge C18 columns (100 × 19 mm, 5 µm particle size) eluting with 0.1% formic acid in water and acetonitrile (95:5 -> 5:95) as the mobile phase.

### 3.2 Synthesis of 4-phenylazetidin-2-one (**4**)



3-amino-3-phenyl-propanoic acid (100 mg, 0.61 mmol, 1 equ) was dissolved in MeCN (60 ml) and methanesulfonyl chloride (277 mg, 2.42 mmol, 4 equ) and sodium hydrogen carbonate (305 mg, 3.63 mmol, 6 equ) were added. The mixture was heated to 60 °C and stirred overnight. Water (10 ml) was added and the mixture extracted 3x with 30 ml ethyl acetate. The combined organics were dried over MgSO_4_, passed through a phase separator and evaporated to dryness. The residue was purified by flash chromatography eluting with a gradient of 0–100% ethyl acetate in heptane to give 4-phenylazetidin-2-one (80 mg, 90%) as a white solid.

^1^H (400 MHz, CDCl_3_): δ 7.39–7.28 (5H, m, H1, H2, H3), 6.71 (1H, brs, H8), 4.70 (1H, dd, *J* = 5.3, 2.5 Hz, H5), 3.41 (1H, ddd, *J* = 14.9, 5.3, 2.5 Hz, H6a), 2.84 (1H, ddd, *J* = 14.9, 2.5, 0.9 Hz, H6b);

^13^C (100 MHz, CDCl_3_): δ 168.46 (C7), 140.33 (C4), 128.87 (*C*2), 128.21 (C1), 125.69 (C3), 50.42 (C5), 47.94 (C6).

Analysis is in agreement with the literature.^18^

### 3.3 Synthesis of 1-phenylazetidin-2-one (**6**)



To a suspension of potassium carbonate (445 mg, 3.22 mmol, 1.2 equ) in DCM (10 ml) was added aniline (250 mg, 2.68 mmol, 1 equ). The mixture was cooled to 0 °C and 3-bromopropanoyl chloride (552 mg, 3.22 mmol, 1 equ) added dropwise. The reaction was stirred at 0 °C for 15 min then allowed to warm up to room temperature. After three hours LCMS showed complete conversion of aniline to the intermediate so 5 ml water was added and the layers separated. The aqueous layer was extracted 3× with 15 ml ethyl acetate and the combined organic layers were dried over MgSO_4_, passed through a phase separator and evaporated to dryness. The residue was recrystallised from 5 ml 1:1 heptane: ethyl acetate to give the intermediate (520 mg, 85%) as a white powder.

MS (ESI) *m*/*z* 228.1 [M+H]^+^

This intermediate was dissolved in DMF (10 ml) and cooled to 0 °C. Sodium *tert*-butoxide (256 mg, 2.68 mmol, 1.2 equ) was added and the reaction was allowed to slowly warm up to room temperature for three hours. The solvent was removed *in vacuo* and the residue was dissolved in 10 ml DCM, washed with 10 ml water and the aqueous layer extracted 2× with 15 ml DCM. The combined organics were dried over MgSO_4_, passed through a phase separator and evaporated to dryness. The residue was purified by flash chromatography eluting with a gradient of 0–80% ethyl acetate in heptane to give 1-phenylazetidin-2-one (95 mg, 24%) as a white powder.

^1^H (500 MHz, CDCl_3_): δ 7.31 (4H, m, H2, H3), 7.06 (1H, m, H1), 3.56 (2H, t, *J* = 4.5 Hz, H5), 3.05 (2H, t, *J* = 4.5 Hz, H6);

^13^C (125 MHz, CDCl_3_): δ 164.54 (C7), 138.51 (C4), 129.12 (*C*2), 123.81 (C1), 116.12 (C3), 37.99 (C5), 36.02 (C6);

MS (ESI) *m*/*z* 148.1 [M+H]^+^

HRMS *m*/*z* (ESI^+^) calcd for C_9_H_10_NO [M+H]^+^: 148.0757, found 148.0464 (6.5 ppm).

Analysis is in agreement with the literature.^19^

### 3.4 Synthesis of (E)-4-(dimethylamino)-*N*-phenyl-but-2-enamide (**10**)



(E)-4-(dimethylamino)but-2-enoic acid hydrochloride (133.4 mg, 0.805 mmol, 1.5 equ) was dissolved in THF (5 ml) with triethylamine (217.3 mg, 2.15 mmol, 4 equ) and T3P (683 mg, 1.07 mmol, 2 equ) (50% in Ethyl acetate (0.6400 ml)) was added. After 15 min aniline (50 mg, 0.54 mmol, 1 equ) was added and the reaction stirred overnight. Then 10 ml saturated sodium hydrogen carbonate solution was added and the solution extracted 3× with 10 ml DCM. The combined organic layers were dried over MgSO_4_, passed through a phase separator and evaporated to dryness. The residue was purified by flash chromatography eluting with a gradient of 0–9% methanol in DCM to give a mixture containing (E)-4-(dimethylamino)-*N*-phenyl-but-2-enamide (46 mg) as a pale yellow powder. This was purified by HPLC eluting with 5–95% acetonitrile in water (0.1% NH4) to give (E)-4-(dimethylamino)-*N*-phenyl-but-2-enamide (41 mg, 37%) as a white powder.

^1^H (400 MHz, CDCl_3_): δ 7.92 (1H, brs, H5), 7.57 (2H, d, *J* = 7.7 Hz, H3), 7.28 (2H, t, *J* = 7.9 Hz, H2), 7.08 (1H, t, *J* = 7.3 Hz, H1), 6.94 (1H, dt, *J* = 15.3, 6.1 Hz, H8), 6.14 (1H, dt, *J* = 15.3, 1.6 Hz, H7), 3.04 (2H, dd, *J* = 6.1, 1.5 Hz, H9), 2.23 (6H, s, *H*10);

^13^C (100 MHz, CDCl_3_): δ 142.83 (C8), 129.20 (*C*2), 125.76 (C7), 124.56 (C1), 119.95 (C3), 60.53 (C9), 45.71 (C10)

HRMS *m*/*z* (ESI^+^) calcd for C_12_H_17_N_2_O [M+H]^+^: 205.1335, found 205.1350 (4.6 ppm).

### 3.5 Synthesis of 1-phenylprop-2-yn-1-ol (**17**)



Benzaldehyde (200 mg, 1.88 mmol, 1 equ) was dissolved in THF (20 ml) and bromo(ethynyl)magnesium (292 mg, 2.26 mmol, 1.2 equ) (0.5 M in THF) was added dropwise over 10 min and the reaction stirred at 0 °C for 30 min. The reaction was then allowed to warm up to room temperature and stirred for three hours. 10 ml saturated ammonium chloride solution was added and stirred vigorously. 10 ml Ethyl acetate was added and the layers separated, the aqueous layer was extracted 2× with 10 ml ethyl acetate. The combined organic layers were washed with 10 ml water, 10 ml brine, dried over MgSO_4_ and evaporated to dryness. The residue was purified by flash chromatography with a gradient of 0–40% ethyl acetate in heptane to give 1-phenylprop-2-yn-1-ol (204 mg, 82%) as a colourless oil.

^1^H (400 MHz, CDCl_3_): δ 7.55 (2H, m, H2), 7.38 (3H, m, H1, H3), 5.43 (1H, dd, *J* = 5.0 2.0 Hz, H5), 3.42 (1H, d, *J* = 5.2 Hz, OH), 2.68 (1H, d, *J* = 2.3 Hz, H7).

Analysis is in agreement with the literature.^20^

### 3.6 Synthesis of 1-phenylprop-2-yn-1-one (**11**)



1-phenylprop-2-yn-1-ol **(17)** (191 mg, 1.45 mmol, 1 equ) was dissolved in DCM (15 ml) and cooled to 0 °C. Dess-Martin (674 mg, 1.59 mmol, 1.1 equ) was added and the reaction stirred while slowly warming up to room temperature. When TLC indicated the reaction was complete 20 ml saturated sodium thiosulfate solution was added, the layers separated and the organic layer washed with 20 ml saturated sodium thiosulfate solution. The aqueous layers were combined and extracted 2× with 15 ml DCM. The combined organics were washed with saturated sodium hydrogen carbonate solution, brine, dried over MgSO_4_, passed through a phase separator and evaporated to dryness. The residue was purified by flash chromatography eluting with a gradient of 0–50% ethyl acetate in heptane to give 1-phenylprop-2-yn-1-one (127 mg, 68%) as a colourless oil which crystallised on standing.

^1^H (400 MHz, CDCl_3_): δ 8.17 (2H, d, *J* = 7.9 Hz, H3), 7.64 (1H, t, *J* = 7.4 Hz, H1), 7.50 (2H, t, *J* = 7.7 Hz, H2), 3.43 (1H, s, H7);

^13^C (100 MHz, CDCl_3_): δ 177.54 (C5), 136.29 (C4), 134.67 (C1), 129.85 (C3), 128.83 (*C*2), 80.87 (C7), 80.41 (C6).

Analysis is in agreement with the literature.^20^

### 3.7 Synthesis of *N*-phenylprop-2-enamide (**12**)



Aniline (100 mg, 1.07 mmol, 1 equ) and triethylamine (326 mg, 3.22 mmol, 3 equ) were dissolved in DCM (10 ml) and cooled to 0 °C. Prop-2-enoyl chloride (97 mg, 1.07 mmol, 1 equ) was added and the reaction allowed to warm up to room temperature overnight. Methanol (10 ml) was added and stirred for 30 min and the reaction was evaporated to dryness. The residue was purified by flash chromatography eluting with a gradient of 0–50% ethyl acetate in heptane to give *N*-phenylprop-2-enamide (103 mg, 65%) as a white powder.

^1^H (400 MHz, DMSO‑*d*_6_): δ 10.09 (1H, brs, H5), 7.66 (2H, d, *J* = 8.2 Hz, H3), 7.32 (2H, t, *J* = 7.7 Hz, H2), 7.06 (1H, t, *J* = 7.3 Hz, H1), 6.44 (1H, dd, *J* = 17.0, 10.1 Hz, H7), 6.25 (1H, dd, *J* = 17.0, 1.8 Hz, H8a), 5.75 (1H, dd, *J* = 10.1, 1.3 Hz, H8b);

^13^C (100 MHz, DMSO‑*d*_6_): δ 163.06 (C6), 138.95 (C4), 131.86 (C7), 128.68 (*C*2), 126.69 (C8), 123.40 (C1), 119.30 (C3);

MS (ESI) *m*/*z* 148.1 [M+H]^+^

HRMS *m*/*z* (ESI^+^) calcd for C_9_H_10_NO [M+H]^+^: 148.0757, found 148.0743 (12.8 ppm).

Analysis is in agreement with the literature.^21^

### 3.8 Synthesis of benzenesulfonyl fluoride (**13**)



Benzenesulfonyl chloride (150 mg, 0.85 mmol, 1 equ) was dissolved in THF (10 ml) and tetrabutylammonium fluoride (444 mg, 1.7 mmol, 2 equ) was added. The reaction was stirred at room temperature overnight. 5 ml saturated sodium hydrogen carbonate solution and 10 ml water was added and the reaction extracted 3× with 20 ml DCM. The combined organics were dried over MgSO_4_ and evaporated to dryness. The residue was purified by flash chromatography eluting with a gradient of 0–80% ethyl acetate in heptane to give benzenesulfonyl fluoride (66 mg, 49%) as a colourless oil.

^1^H (500 MHz, CDCl_3_): δ 8.01 (2H, m, H3), 7.79 (1H, m, H1), 7.64 (2H, m, H2);

^13^C (125 MHz, CDCl_3_): δ 135.71 (C1), 133.24 (d, *J*_C-F_ = 24.4 Hz, C4), 129.80 (*C*2), 128.52 (C3).

Analysis is in agreement with the literature.^22^

### 3.9 Synthesis of methyl *N*-phenylcarbamate (**14**)



Aniline (200 mg, 2.15 mmol, 1 equ) was dissolved in DCM (25 ml) and cooled to 0 °C. Pyridine (221 mg, 2.79 mmol, 1.3 equ) was added followed by methyl chloroformate (244 mg, 2.58 mmol, 1.2 equ) and the reaction stirred while slowly warming up to room temperature. When TLC indicated the reaction was complete 10 ml water was added, the layers separated and the aqueous layer extracted 2× with 15 ml DCM. The combined organics were passed through a phase separator and evaporated to dryness. The residue was purified by flash chromatography eluting with a gradient of 0–50% ethyl acetate in heptane to give methyl *N*-phenylcarbamate (325 mg, 100%) as a pale yellow oil.

^1^H (400 MHz, CDCl_3_): δ 7.38 (2H, d, *J* = 8.0 Hz, H3), 7.31 (2H, t, *J* = 7.8 Hz, H2), 7.07 (1H, t, *J* = 7.3 Hz, H1), 6.63 (1H, brs, H5), 3.78 (3H, s, H7);

^13^C (100 MHz, CDCl_3_): δ 154.19 (C6), 138.00 (C4), 129.20 (*C*2), 123.64 (C1), 118.92 (C3), 52.45 (C7);

HRMS *m*/*z* (ESI^+^) calcd for C_8_H_07_NO_2_ [M+H]^+^: 152.0706, found 152.0716 (5.9 ppm).

Analysis is in agreement with the literature.^23^

### 3.10 Synthesis of *N*-phenylethenesulfonamide (**15**)



Aniline (100 mg, 1.074 mmol, 1 equ) and 2-chloroethanesulfonyl chloride (175 mg, 1.074 mmol, 1 equ) were dissolved in DCM (10 ml) and cooled to 0 °C. Triethylamine (326 mg, 3.22 mmol, 3 equ) was added and the reaction was stirred for 10 min then allowed to warm up to room temperature. After 2 h TLC suggested no starting material remained so the reaction was quenched with 20 ml water and the layers separated. The aqueous layer was extracted 2× with 20 ml DCM, the combined organics washed with 20 ml 1 N HCl, 20 ml brine, dried over MgSO_4_ and evaporated to dryness. The residue was purified by flash chromatography eluting with a gradient of 0–50% ethyl acetate in heptane to give a colourless oil. This was dissolved in 5 ml DCM and 5 ml heptane was added, the DCM was removed under reduced pressure. A precipitate formed and was isolated by filtration to give *N*-phenylethenesulfonamide (196 mg, 100%) as off white crystals.

^1^H (500 MHz, CDCl_3_): δ 7.45 (1H, brs, H5), 7.30 (2H, m, H2), 7.21 (2H, m, H3), 7.14 (1H, m, H1), 6.58 (1H, dd, *J* = 16.5, 10.0 Hz, H6), 6.24 (1H, d, *J* = 16.6 Hz, H7a), 5.91 (1H, d, *J* = 10.0 Hz, H7b);

^13^C (100 MHz, CDCl_3_): δ 137.76 (C4), 136.25 (C6), 129.14 (*C*2), 127.55 (C7), 123.80 (C1), 119.70 (C3);

MS (ESI) *m*/*z* 184.1 [M+H]^+^

HRMS *m*/*z* (ESI^+^) calcd for C_8_H_10_NO_2_S [M+H]^+^: 184.0427, found 184.0430 (5.3 ppm).

Analysis is in agreement with the literature.^24^

### 3.11 Synthesis of 1-phenylprop-2-en-1-ol (**18**)



Benzaldehyde (100 mg, 0.94 mmol, 1 equ) was dissolved in THF (10 ml) and bromo(vinyl)magnesium (148 mg, 1.13 mmol, 1.2 equ) (1 M in THF) was added dropwise over 10 min and the reaction stirred at 0 °C for 30 min. The reaction was then allowed to warm up to room temperature and stirred overnight. Then 10 ml saturated ammonium chloride solution was added and stirred vigorously. 10 ml ethyl acetate was added and the layers separated, the aqueous layer was extracted 2× with 10 ml ethyl acetate. The combined organic layers were washed with 10 ml water, 10 ml brine, dried over MgSO_4_ and evaporated to dryness. The residue was purified by flash chromatography eluting with a gradient of 0–80% ethyl acetate in heptane to give 1-phenylprop-2-en-1-ol (118 mg, 93%).

^1^H (400 MHz, CDCl_3_): δ 7.23 (4H, d, *J* = 4.4 Hz, H2, H3), 7.17 (1H, m, H1), 5.90 (1H, ddd, *J* = 17.1, 10.3, 6.0 Hz, H6), 5.20 (1H, dt, *J* = 17.1, 1.4 Hz, H7a), 5.05 (1H, dt, *J* = 10.3, 1.3 Hz, H7b), 5.02 (1H, d, *J* = 5.9 Hz, H5).

Analysis is in agreement with the literature.^25^

### 3.12 Synthesis of 1-phenylprop-2-en-1-one (**16**)



1-phenylprop-2-en-1-ol **(18)** (216 mg, 1.61 mmol, 1 equ) was dissolved in DCM (15 ml) and cooled to 0 °C. Dess-Martin (751 mg, 1.78 mmol, 1.1 equ) was added and the reaction stirred while slowly warming up to room temperature.

When TLC indicated the reaction was complete 20 ml saturated sodium thiosulfate solution was added, the layers separated and the organic layer washed with 20 ml saturated sodium thiosulfate solution. The aqueous layers were combined and extracted 2× with 15 ml DCM. The combined organics were washed with saturated sodium hydrogen carbonate solution, brine, dried over MgSO_4,_ passed through a phase separator and evaporated to dryness.

The residue was purified by flash chromatography eluting with a gradient of 0–50% ethyl acetate in heptane to give 1-phenylprop-2-en-1-one (200 mg, 94%) as a colourless oil. Despite the product being stored in the freezer it was observed to have turned brown when needed so was repurified by flash chromatography (12 g column) eluting with a gradient of 0–2% methanol in DCM to give 1-phenylprop-2-en-1-one (43 mg, 20%) yield as a colourless oil.

^1^H (400 MHz, CDCl_3_): δ 7.93 (2H, m, H3), 7.55 (1H, m, H1), 7.45 (2H, m, H2), 7.14 (1H, dd, *J* = 17.1, 10.6 Hz, H6), 6.42 (1H, dd, *J* = 17.1, 1.7 Hz, H7a), 5.90 (1H, dd, *J* = 10.6, 1.7 Hz, H7b);

^13^C (100 MHz, CDCl_3_): δ 191.07 (C5), 137.27 (C4), 133.03 (C1), 132.38 (C6), 130.22 (C7), 128.72 (*C*2), 128.65 (C3).

Analysis is in agreement with the literature.^26^

### 3.13 Synthesis of Boc-Arg-OMe (**19**)



2-(*tert*-butoxycarbonylamino)-5-guanidino-pentanoic acid (100 mg, 0.36 mmol, 1 equ) and N,N′-dicyclohexylmethanediimine (83 mg, 0.40 mmol, 1.1 equ) were dissolved in DCM (4 ml) with N,*N*-dimethylpyridin-4-amine (4.5 mg, 0.04 mmol, 0.1 equ). Methanol (74 µL, 1.83 mmol, 5 equ) was added and the reaction stirred for five days. LCMS indicated the reaction was complete so it was filtered and evaporated to dryness. The residue was purified by flash chromatography eluting with a gradient of 0–10% methanol in DCM followed by HPLC 5–95% acetonitrile in water (0.1% HCOOH) to give methyl 2-(*tert*-butoxycarbonylamino)-5-guanidino-pentanoate (56 mg, 53%) as a colourless oil.

^1^H (400 MHz, CDCl_3_): δ 8.19 (1H, brs, NH), 7.18 (4H, brs, NHs), 4.21 (1H, brs, H3), 3.73 (3H, s, H1), 3.30 (2H, brs, H6a, NH), 3.19 (1H, m, H6b), 1.84 (1H, brs, H4a), 1.69 (3H, brs, H4b, H5), 1.41 (9H, s, H14);

^13^C (100 MHz, CDCl_3_): δ 169.64 (C2), 157.75 (C8), 156.37 (C12), 80.39 (H13), 52.99 (C3), 52.69 (C1), 40.93 (C6), 29.92 (C4), 28.49 (C14), 25.07 (C5);

HRMS *m*/*z* (ESI^+^) calcd for C_12_H_25_N_4_O_4_ [M+H]^+^: 289.1870, found 289.1874 (1.3 ppm).

### 3.14 NMR assay

To 0.5 ml of the assay buffer (100 mM PBS in 90% H_2_O:10% D_2_O) was added 5 μl of the covalent warhead (0.2 M in DMSO‑*d*_6_), in an NMR tube and a ^1^H NMR spectra was acquired. To 0.5 ml of the same buffer was added 5 μl of the amino acid (2 M in DMSO‑*d*_6_). This was added to the NMR tube and the solution mixed by inverting the tube several times. If necessary, sonication was used to ensure complete dissolution. This gave a final solution of 1 mM covalent warhead, 10 mM amino acid in 100 mM PBS. A ^1^H NMR was typically recorded on this sample every 10 min for 6 h. In the experiment, there was a ∼4 s data acquisition time, followed by a 4 s relaxation delay to minimise peak saturation. Each data point required approximately 2 min (16 scans per data point).
