# Supplementary material for: Characterising covalent warhead reactivity
Source: Bioorg Med Chem. 2019 May 15;27(10):2066–74. doi: 10.1016/j.bmc.2019.04.002 (PMC6538824; doi:10.1016/j.bmc.2019.04.002)
Supplement: Supplementary data 1 [file mmc1.pdf]

4-phenylazetidin-2-one (4)

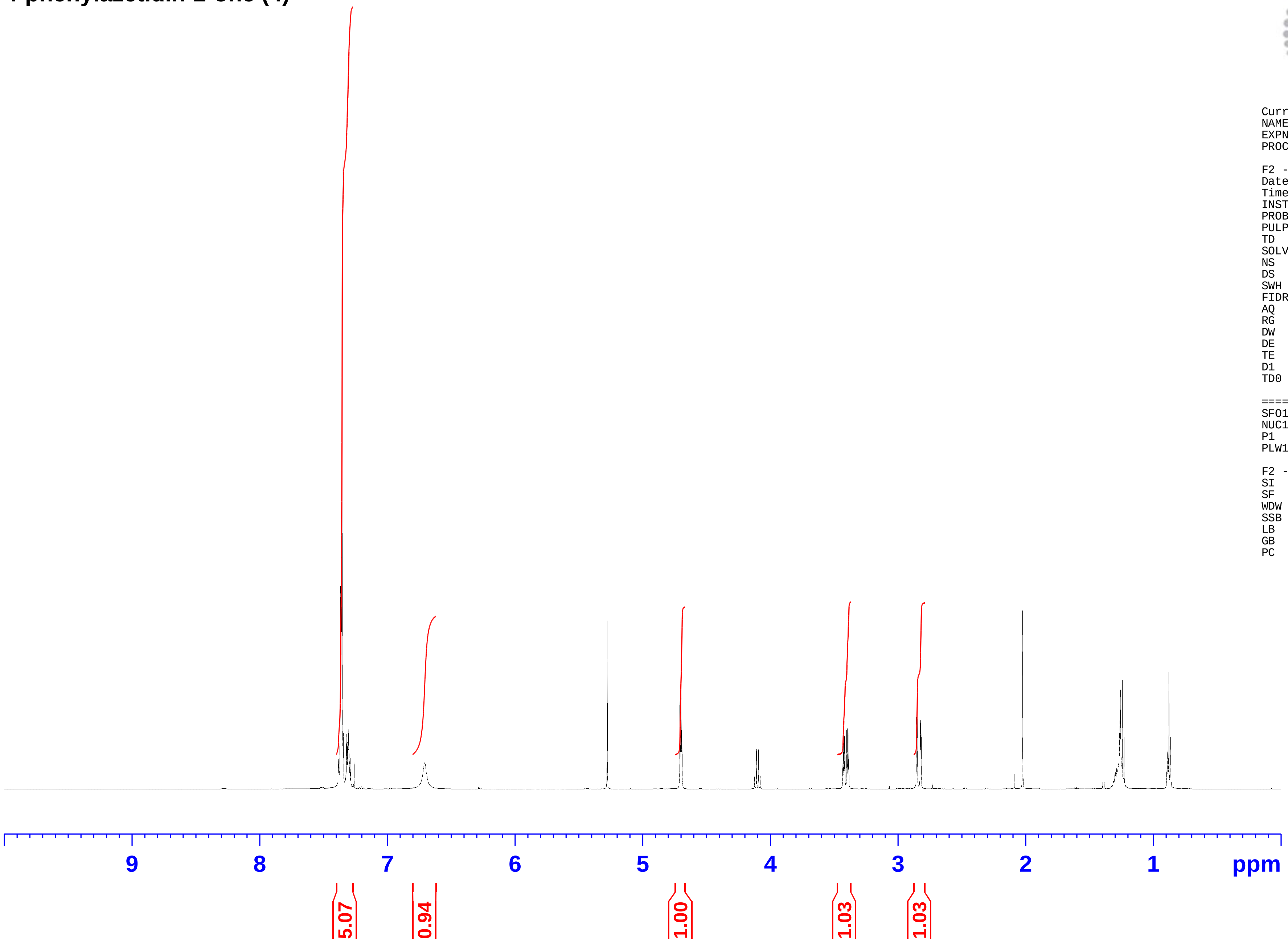


```
Current Data Parameters
NAME
EXPNO                  1
PROCNO                 1

F2 - Acquisition Parameters
Date_           20170503
Time               15.38
INSTRUM            spect
PROBHD   5 mm QNP 1H/13
PULPROG             zg30
TD                 65536
SOLVENT            CDCl3
NS                    16
DS                     2
SWH           10000.000 Hz
FIDRES         0.152588 Hz
AQ            3.2767999 sec
RG                   114
DW                50.000 usec
DE                  6.50 usec
TE                 298.2 K
D1            1.00000000 sec
TD0                    1

======== CHANNEL f1 ========
SFO1         500.1330885 MHz
NUC1                  1H
P1                 10.00 usec
PLW1         25.00000000 W

F2 - Processing parameters
SI                 65536
SF           500.1300127 MHz
WDW                   EM
SSB      0
LB                  0.30 Hz
GB       0
PC                  1.00
```

4-phenylazetidin-2-one (4)

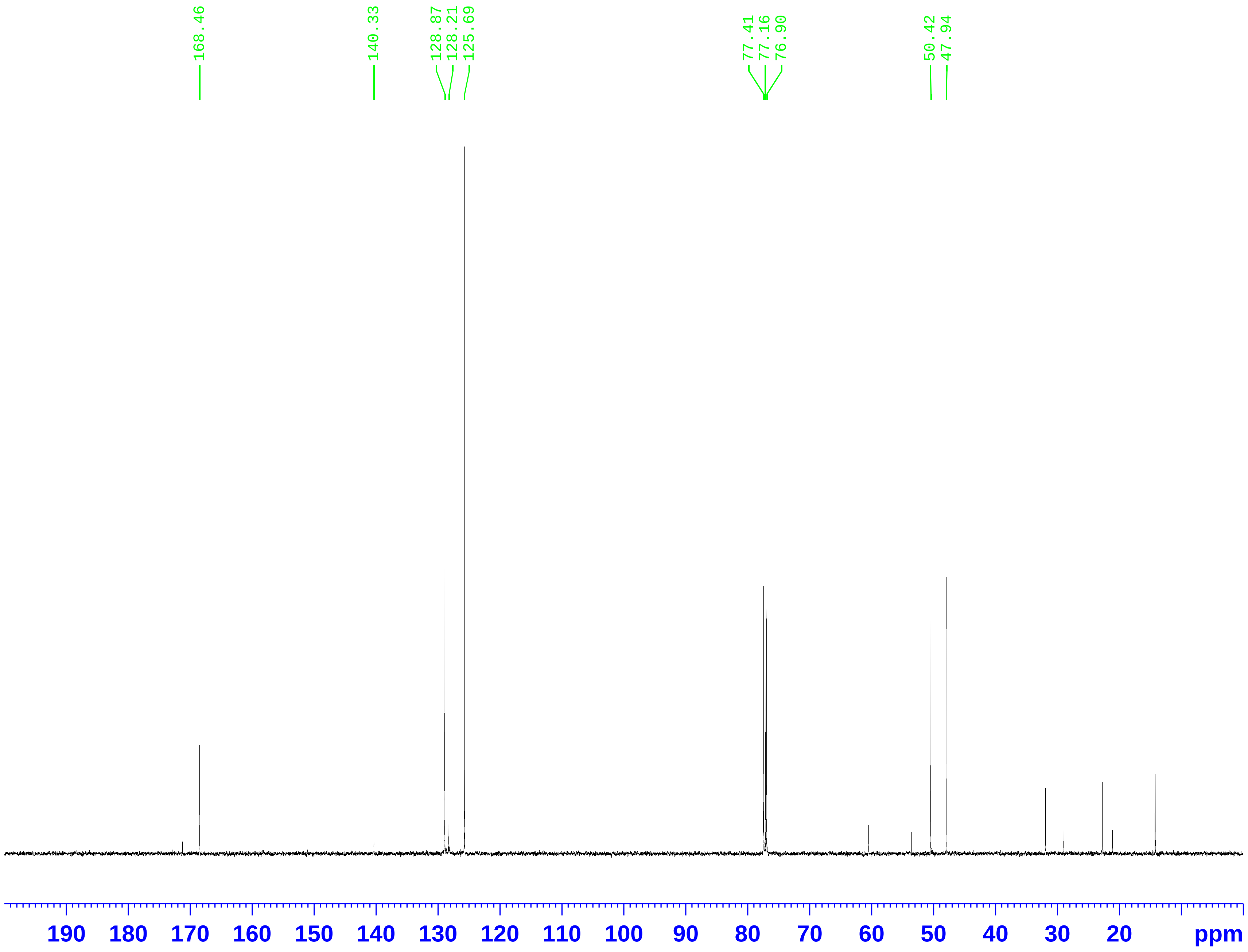


```
Current Data Parameters
NAME
EXPNO                    2
PROCNO                   1

F2 - Acquisition Parameters
Date_             20170503
Time                 16.00
INSTRUM              spect
PROBHD   5 mm QNP 1H/13
PULPROG            zgpg30
TD                   65536
SOLVENT              CDCl3
NS                     256
DS                       4
SWH            29761.904 Hz
FIDRES          0.454131 Hz
AQ             1.1010048 sec
RG                    2050
DW                  16.800 usec
DE                    6.50 usec
TE                   298.2 K
D1            2.00000000 sec
D11           0.03000000 sec
TD0                      1

======== CHANNEL f1 ========
SFO1         125.7703637 MHz
NUC1                   13C
P1                    7.50 usec
PLW1         92.00000000 W

======== CHANNEL f2 ========
SFO2         500.1320005 MHz
NUC2                    1H
CPDPRG[2           waltz16
PCPD2                80.00 usec
PLW2         25.00000000 W
PLW12         0.39063001 W
PLW13         0.19648001 W

F2 - Processing parameters
SI                   32768
SF           125.7577833 MHz
WDW                     EM
SSB       0
LB                    1.00 Hz
GB        0
PC                    1.40
```

1-phenylazetidin-2-one (6)

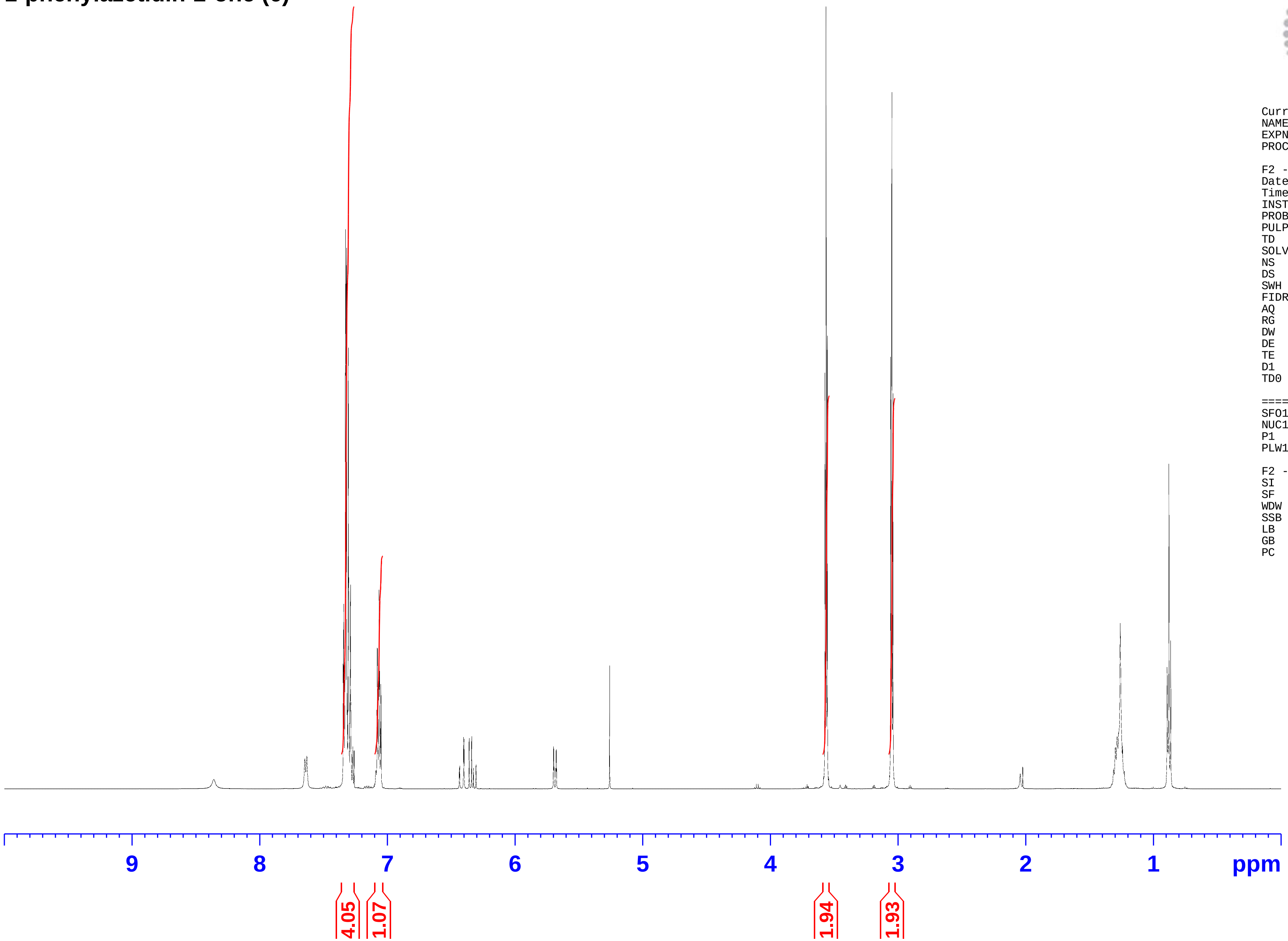


```
Current Data Parameters
NAMEI
EXPNO                    1
PROCNO                   1

F2 - Acquisition Parameters
Date_             20170720
Time                 15.37
INSTRUM              spect
PROBHD   5 mm QNP 1H/13
PULPROG               zg30
TD                   65536
SOLVENT              CDCl3
NS                      16
DS                       2
SWH             10000.000 Hz
FIDRES           0.152588 Hz
AQ              3.2767999 sec
RG                    71.8
DW                  50.000 usec
DE                    6.50 usec
TE                   298.2 K
D1              1.00000000 sec
TD0                      1

======== CHANNEL f1 ========
SFO1          500.1330885 MHz
NUC1                    1H
P1                   10.00 usec
PLW1           25.00000000 W

F2 - Processing parameters
SI                   65536
SF            500.1300126 MHz
WDW                     EM
SSB       0
LB                    0.30 Hz
GB        0
PC                    1.00
```

1-phenylazetidin-2-one (6)

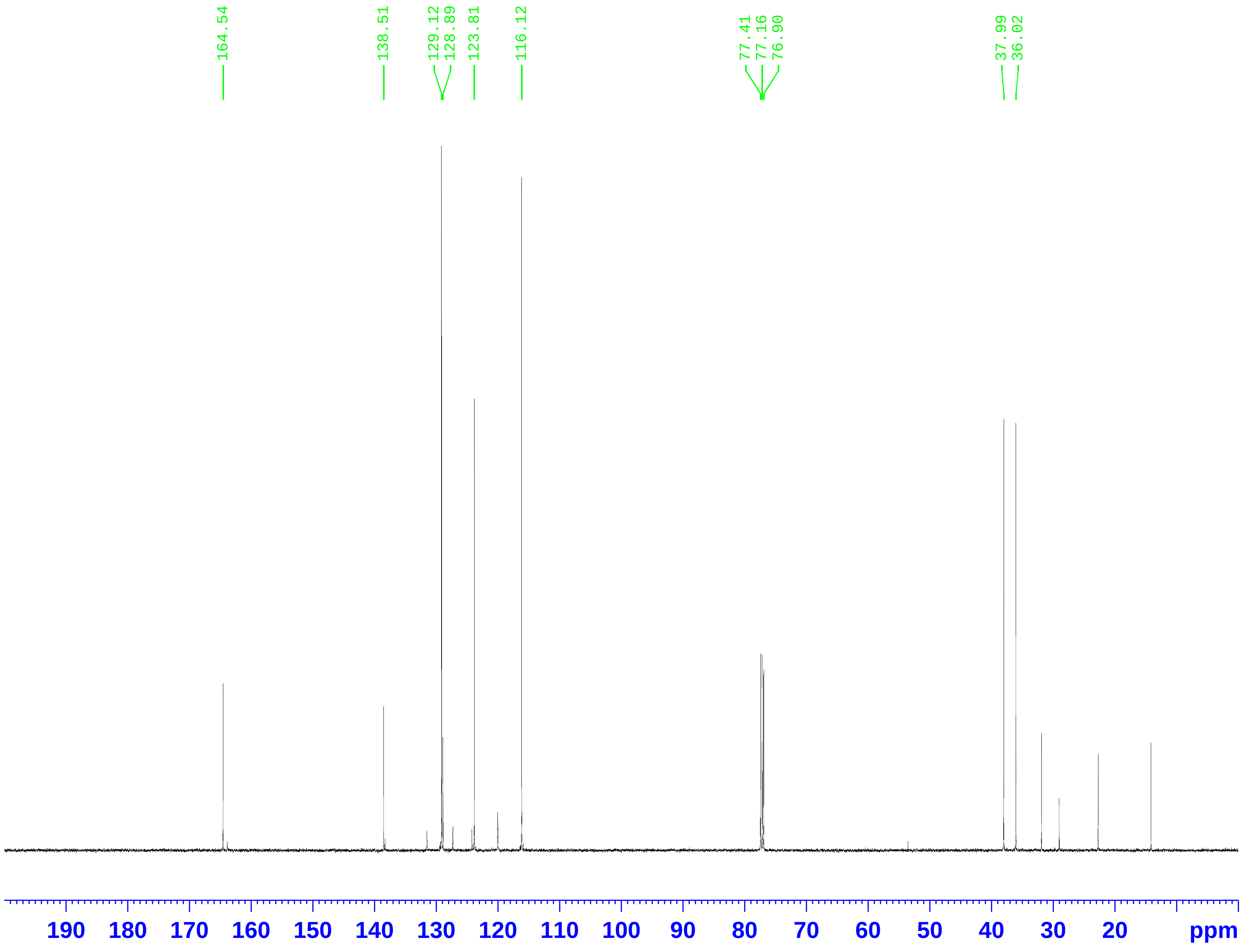


```
Current Data Parameters
NAME
EXPNO                    3
PROCNO                   1

F2 - Acquisition Parameters
Date_             20170720
Time                 16.05
INSTRUM              spect
PROBHD   5 mm QNP 1H/13
PULPROG            zgpg30
TD                   65536
SOLVENT              CDCl3
NS                     256
DS                       4
SWH             29761.904 Hz
FIDRES           0.454131 Hz
AQ              1.1010048 sec
RG                    2050
DW                  16.800 usec
DE                    6.50 usec
TE                   298.2 K
D1            2.00000000 sec
D11           0.03000000 sec
TD0                      1

======== CHANNEL f1 ========
SFO1          125.7703637 MHz
NUC1                   13C
P1                    7.50 usec
PLW1          92.00000000 W

======== CHANNEL f2 ========
SFO2          500.1320005 MHz
NUC2                    1H
CPDPRG[2           waltz16
PCPD2                80.00 usec
PLW2          25.00000000 W
PLW12          0.39063001 W
PLW13          0.19648001 W

F2 - Processing parameters
SI                   32768
SF            125.7577904 MHz
WDW                     EM
SSB      0
LB                    1.00 Hz
GB       0
PC                    1.40
```

(E)-4-(dimethylamino)-N-phenyl-but-2-enamide (10)

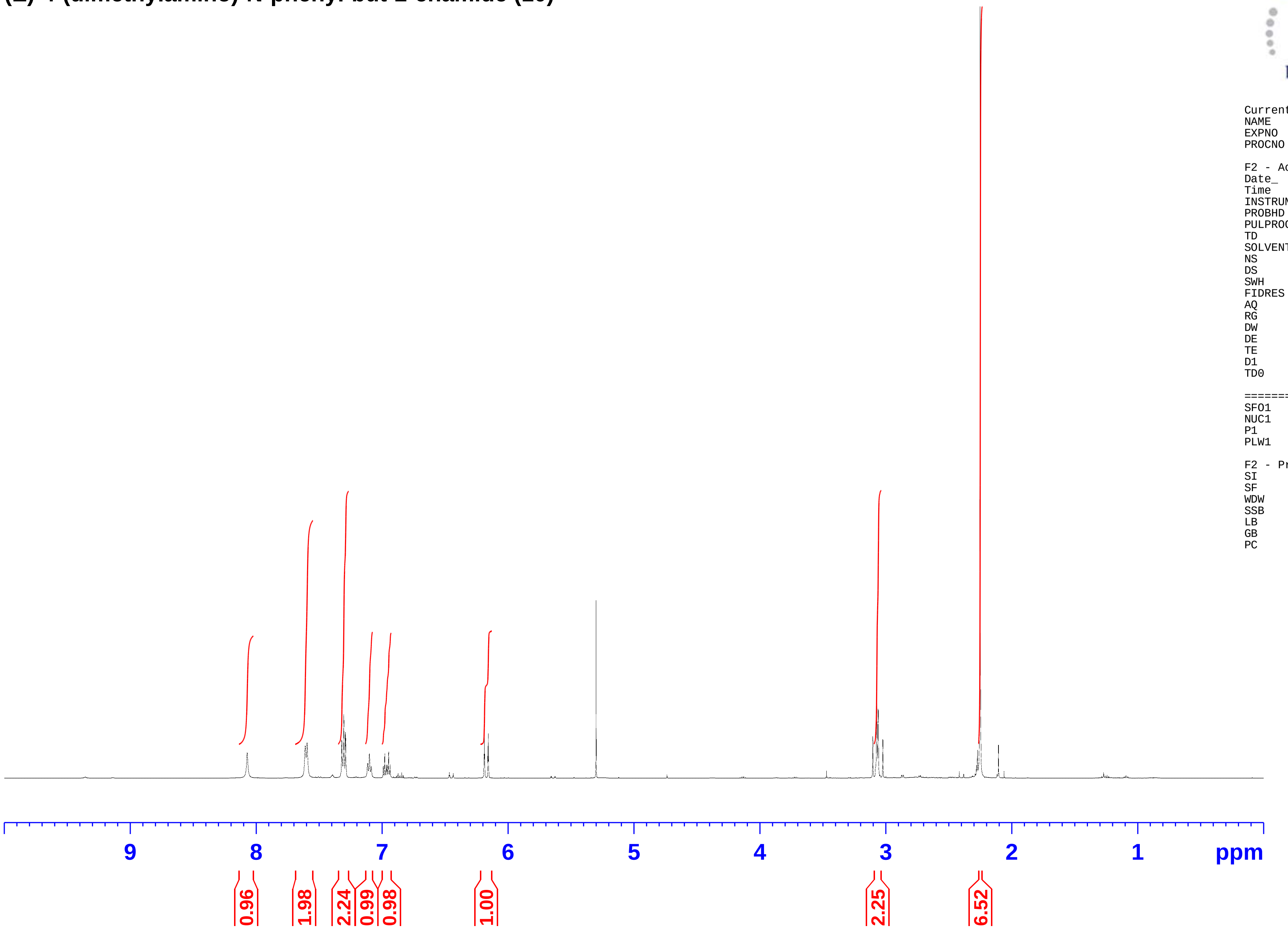

UNIVERSITY OF DUNDEE

```
Current Data Parameters
NAME
EXPNO                    1
PROCNO                   1

F2 - Acquisition Parameters
Date_             20170420
Time                 11.56
INSTRUM              spect
PROBHD   5 mm QNP 1H/13
PULPROG               zg30
TD                   65536
SOLVENT              CDCl3
NS                      16
DS                       2
SWH          10000.000 Hz
FIDRES        0.152588 Hz
AQ           3.2767999 sec
RG                     101
DW              50.000 usec
DE                6.50 usec
TE               294.5 K
D1          1.00000000 sec
TD0                      1

======== CHANNEL f1 ========
SFO1       500.1330885 MHz
NUC1                    1H
P1                10.00 usec
PLW1       25.00000000 W

F2 - Processing parameters
SI                   65536
SF         500.1299979 MHz
WDW                     EM
SSB       0
LB                  0.30 Hz
GB        0
PC                    1.00
```

(E)-4-(dimethylamino)-N-phenyl-but-2-enamide (10)

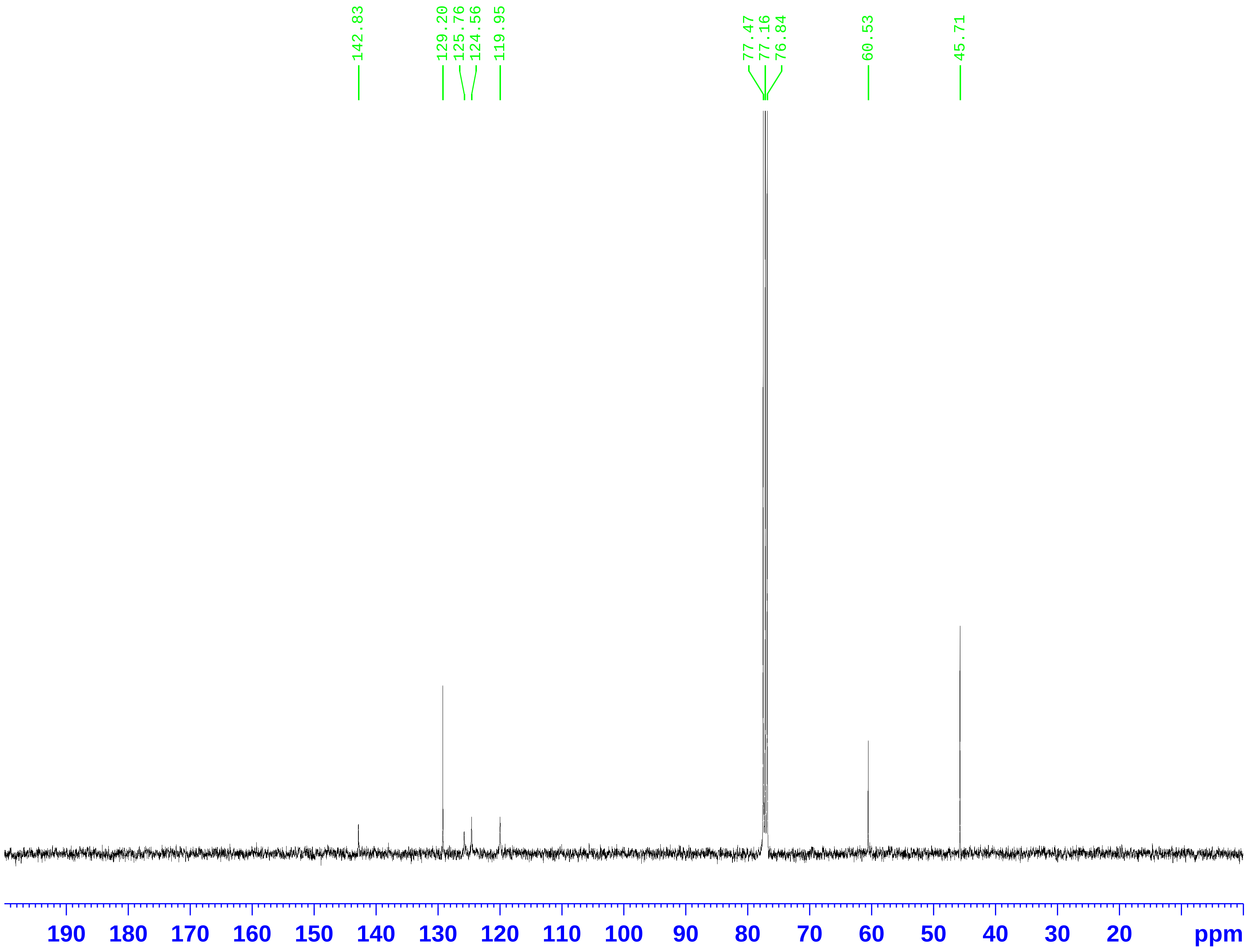


```
Current Data Parameters
NAME1
EXPNO                  23
PROCNO                  1

F2 - Acquisition Parameters
Date_            20181114
Time                 3.06
INSTRUM             spect
PROBHD    5 mm PADUL 13C
PULPROG             udeft
TD                  17996
SOLVENT             CDCl3
NS                   2048
DS                      0
SWH             25000.000 Hz
FIDRES           1.389198 Hz
AQ              0.3599200 sec
RG                 196.14
DW                 20.000 usec
DE                   8.66 usec
TE                  294.6 K
D1             3.00000000 sec
D11            0.03000000 sec
D12            0.00002000 sec
D20          200.00000000 sec
TD0                     1

======== CHANNEL f1 ========
SFO1          100.6238346 MHz
NUC1                  13C
P1                  10.00 usec
P13               2000.00 usec
P26                500.00 usec
PLW1          36.00000000 W
SPNAM[5]      Crp60comp.4
SPOAL5              0.500
SPOFFS5   0 Hz
SPW5           5.50040007 W
SPNAM[8]  Crp60,0.5,20.1
SPOAL8              0.500
SPOFFS8   0 Hz
SPW8           5.50040007 W

======== CHANNEL f2 ========
SFO2          400.1316005 MHz
NUC2                   1H
CPDPRG[2          waltz64
PCPD2               90.00 usec
PLW2          20.00000000 W
PLW12          0.24691001 W

F2 - Processing parameters
SI                 262144
SF            100.6127551 MHz
WDW                    EM
SSB       0
LB                   2.00 Hz
GB        0
PC                   1.40
```

1-phenylprop-2-yn-1-ol (17)

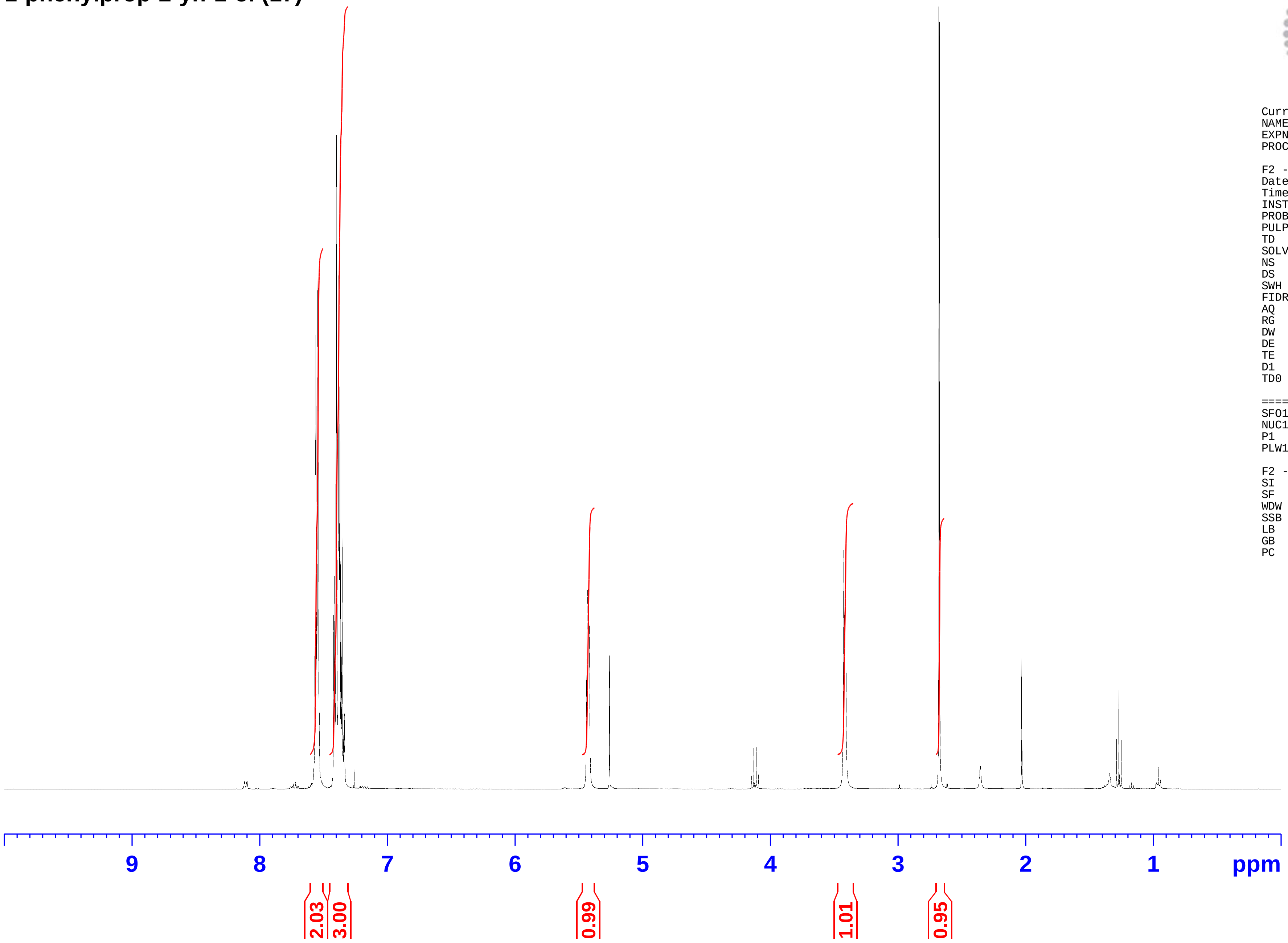

Current Data Parameters

NAME	
EXPNO	1
PROCNO	1

F2 - Acquisition Parameters

Date_	20170524
Time	16.30
INSTRUM	spect
PROBHD	5 mm PADUL 13C
PULPROG	zg30
TD	131072
SOLVENT	CDCl3
NS	16
DS	4
SWH	12019.230 Hz
FIDRES	0.091699 Hz
AQ	5.4525952 sec
RG	14.07
DW	41.600 usec
DE	12.17 usec
TE	298.2 K
D1	0.10000000 sec
TD0	1

======== CHANNEL f1 ========

SFO1	400.1324710 MHz
NUC1	1H
P1	10.00 usec
PLW1	20.00000000 W

F2 - Processing parameters

SI	131072
SF	400.1300093 MHz
WDW	EM
SSB	0
LB	0.10 Hz
GB	0
PC	1.00

1-phenylprop-2-yn-1-one (11)

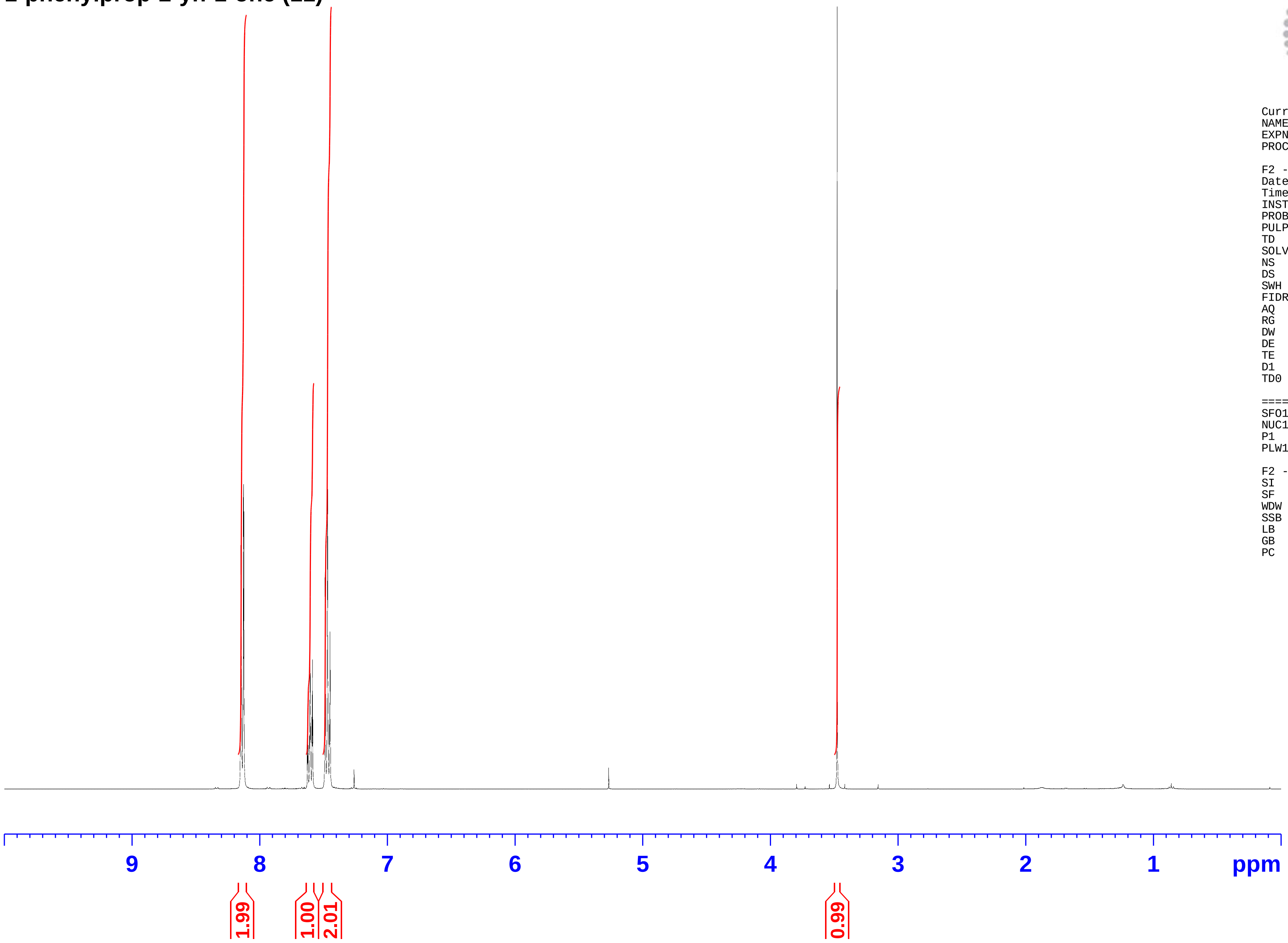


```
Current Data Parameters
NAME
EXPNO                    1
PROCNO                   1

F2 - Acquisition Parameters
Date_             20170530
Time                 13.11
INSTRUM              spect
PROBHD    5 mm PADUL 13C
PULPROG               zg30
TD                  131072
SOLVENT              CDCl3
NS                      16
DS                       4
SWH              12019.230 Hz
FIDRES            0.091699 Hz
AQ               5.4525952 sec
RG                   39.17
DW                  41.600 usec
DE                   12.17 usec
TE                   298.2 K
D1              0.10000000 sec
TD0                      1

======== CHANNEL f1 ========
SFO1           400.1324710 MHz
NUC1                    1H
P1                   10.00 usec
PLW1           20.00000000 W

F2 - Processing parameters
SI                  131072
SF             400.1300092 MHz
WDW                     EM
SSB       0
LB                    0.10 Hz
GB        0
PC                    1.00
```

1-phenylprop-2-yn-1-one (11)

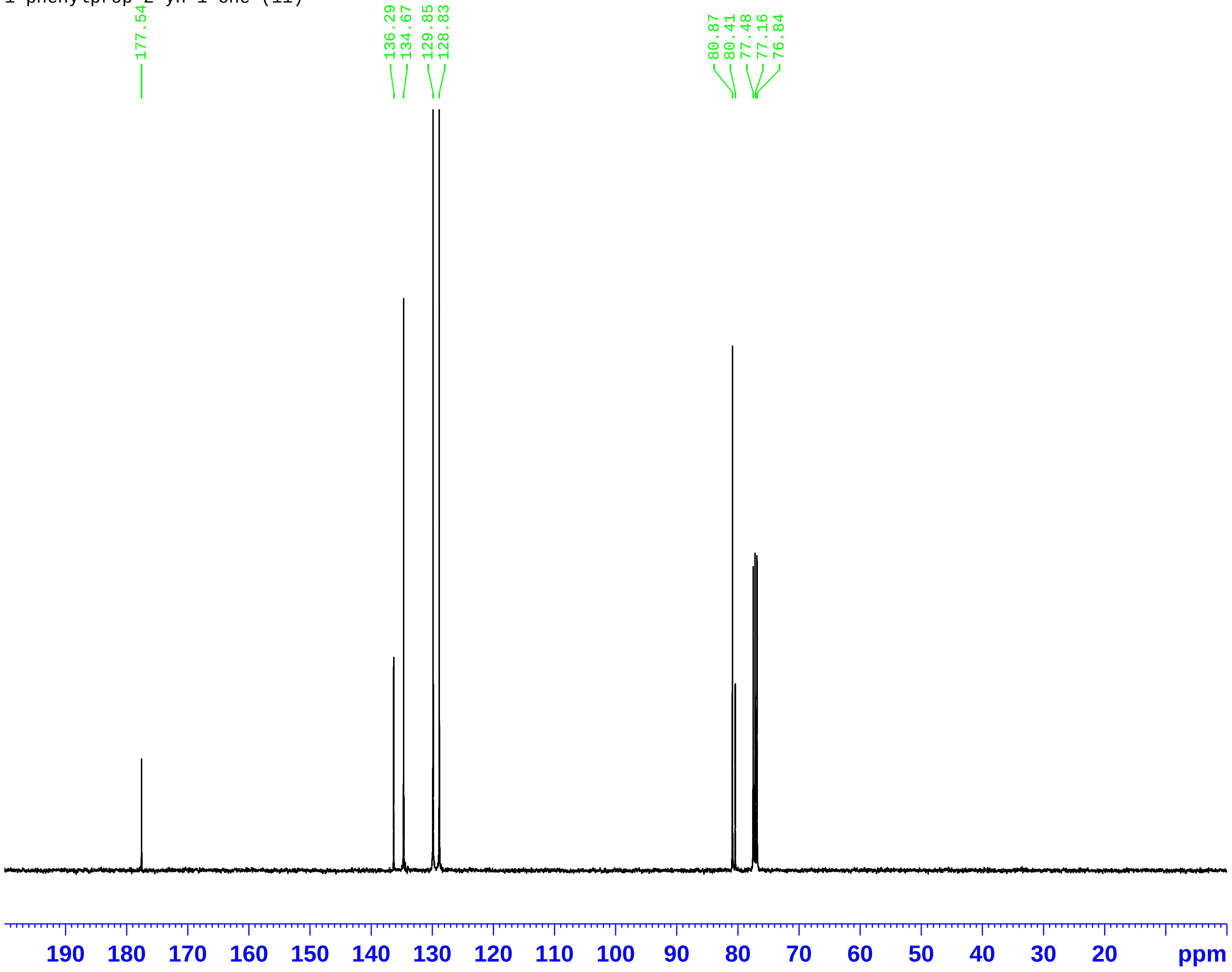


```
Current Data Parameters
NAME
EXPNO                    21
PROCNO                    1

F2 - Acquisition Parameters
Date_              20181114
Time                   0.44
INSTRUM               spect
PROBHD    5 mm PADUL 13C
PULPROG               udeft
TD                    17996
SOLVENT               CDCl3
NS                     2048
DS                        0
SWH           25000.000 Hz
FIDRES         1.389198 Hz
AQ            0.3599200 sec
RG                   196.14
DW               20.000 usec
DE                 8.66 usec
TE                  294.6 K
D1           3.00000000 sec
D11          0.03000000 sec
D12          0.00002000 sec
D20        200.00000000 sec
TD0                       1

======== CHANNEL f1 ========
SFO1        100.6238346 MHz
NUC1                    13C
P1                10.00 usec
P13             2000.00 usec
P26              500.00 usec
PLW1        36.00000000 W
SPNAM[5]     Crp60comp.4
SPOAL5                0.500
SPOFFS5   0 Hz
SPW5         5.50040007 W
SPNAM[8] Crp60,0.5,20.1
SPOAL8                0.500
SPOFFS8   0 Hz
SPW8         5.50040007 W

======== CHANNEL f2 ========
SFO2        400.1316005 MHz
NUC2                     1H
CPDPRG[2            waltz64
PCPD2             90.00 usec
PLW2        20.00000000 W
PLW12        0.24691001 W

F2 - Processing parameters
SI                   262144
SF          100.6127560 MHz
WDW                      EM
SSB       0
LB                  2.00 Hz
GB        0
PC                     1.40
```

N-phenylprop-2-enamide (12)

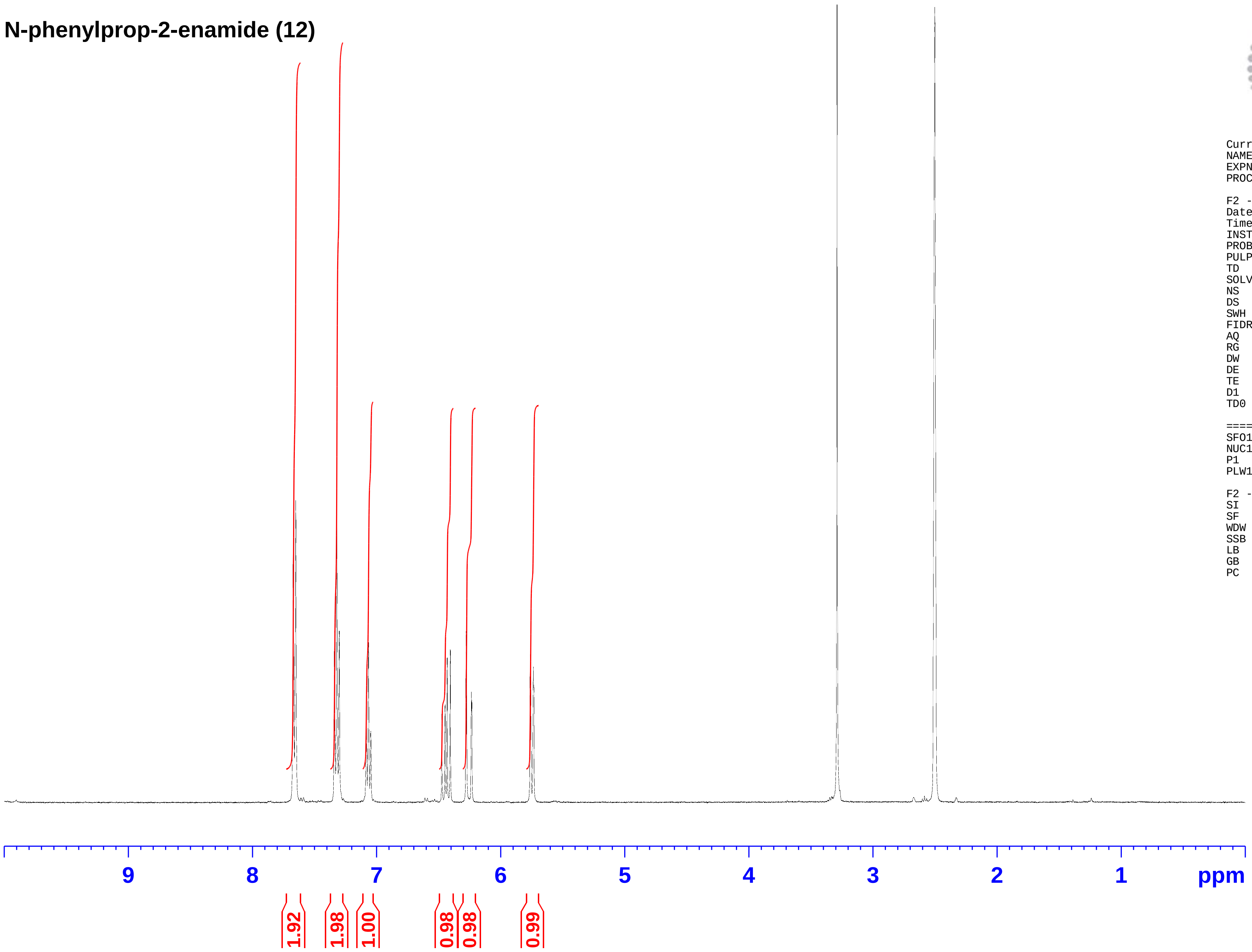

UNIVERSITY OF DUNDEE

```
Current Data Parameters
NAME
EXPNO                  30
PROCNO                  1

F2 - Acquisition Parameters
Date_            20181112
Time                12.32
INSTRUM             spect
PROBHD   5 mm PADUL 13C
PULPROG              zg30
TD                  65536
SOLVENT              DMSO
NS                     16
DS                      2
SWH              8000.000 Hz
FIDRES           0.122070 Hz
AQ              4.0960002 sec
RG                 196.14
DW                 62.500 usec
DE                  11.07 usec
TE                  303.1 K
D1             1.00000000 sec
TD0                     1

======== CHANNEL f1 ========
SFO1          400.1324710 MHz
NUC1                   1H
P1                  10.00 usec
PLW1          20.00000000 W

F2 - Processing parameters
SI                  65536
SF            400.1300028 MHz
WDW                    EM
SSB      0
LB                   0.30 Hz
GB       0
PC                   1.00
```

N-phenylprop-2-enamide (12)

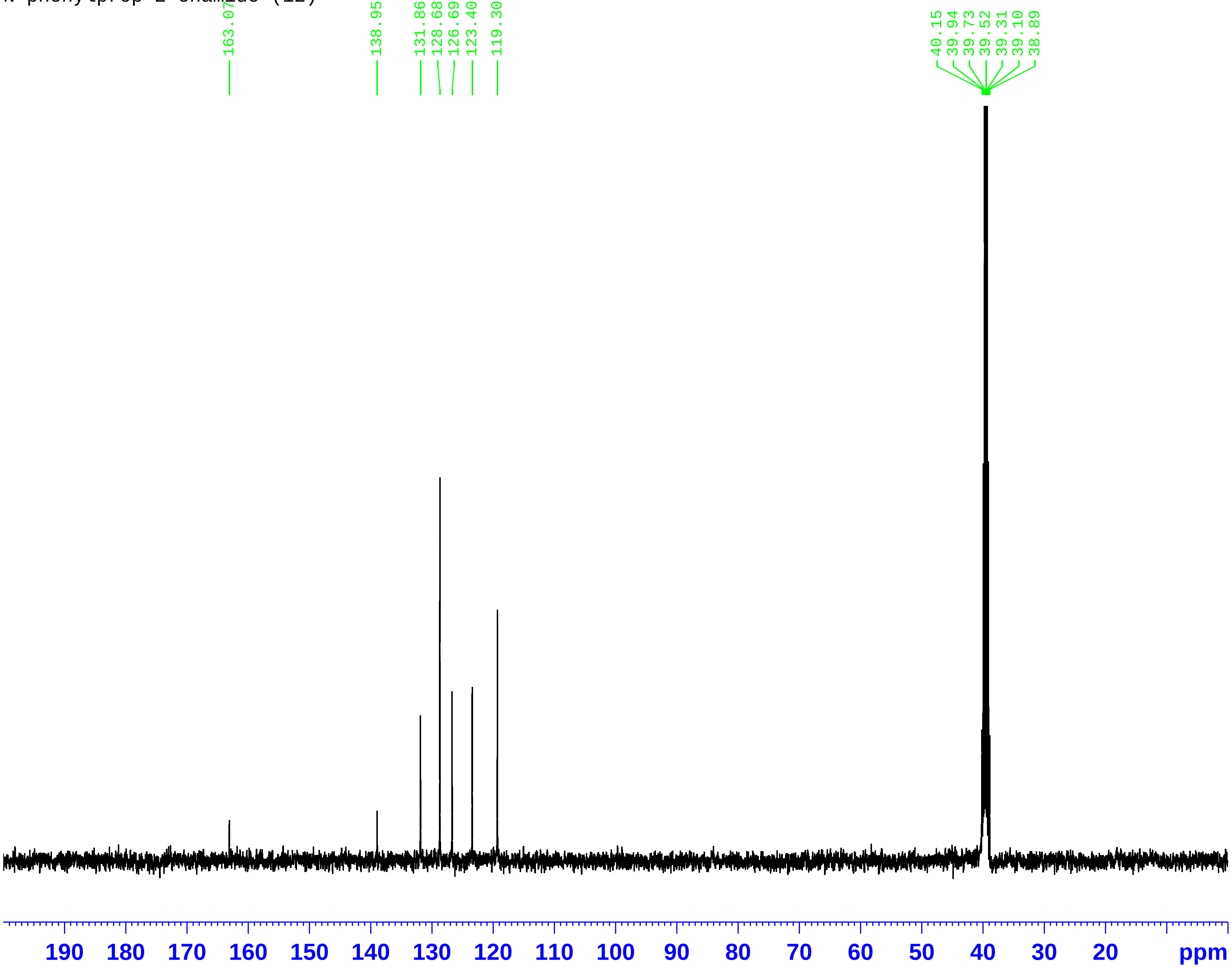

UNIVERSITY OF DUNDEE

Current Data Parameters
NAME
EXPNO 32
PROCNO 1

F2 - Acquisition Parameters
Date_ 20181112
Time 20.12
INSTRUM spect
PROBHD 5 mm PADUL 13C
PULPROG udeft
TD 17996
SOLVENT DMSO
NS 256
DS 0
SWH 25000.000 Hz
FIDRES 1.389198 Hz
AQ 0.3599200 sec
RG 196.14
DW 20.000 usec
DE 8.66 usec
TE 303.1 K
D1 3.00000000 sec
D11 0.03000000 sec
D12 0.00002000 sec
D20 200.00000000 sec
TD0 1

======== CHANNEL f1 ========
SFO1 100.6238346 MHz
NUC1 13C
P1 10.00 usec
P13 2000.00 usec
P26 500.00 usec
PLW1 36.00000000 W
SPNAM[5] Crp60comp.4
SPOAL5 0.500
SPOFFS5 0 Hz
SPW5 5.50040007 W
SPNAM[8] Crp60,0.5,20.1
SPOAL8 0.500
SPOFFS8 0 Hz
SPW8 5.50040007 W

======== CHANNEL f2 ========
SFO2 400.1316005 MHz
NUC2 1H
CPDPRG[2 waltz64
PCPD2 90.00 usec
PLW2 20.00000000 W
PLW12 0.24691001 W

F2 - Processing parameters
SI 262144
SF 100.6128228 MHz
WDW EM
SSB 0
LB 2.00 Hz
GB 0
PC 1.40

benzenesulfonyl fluoride (13)

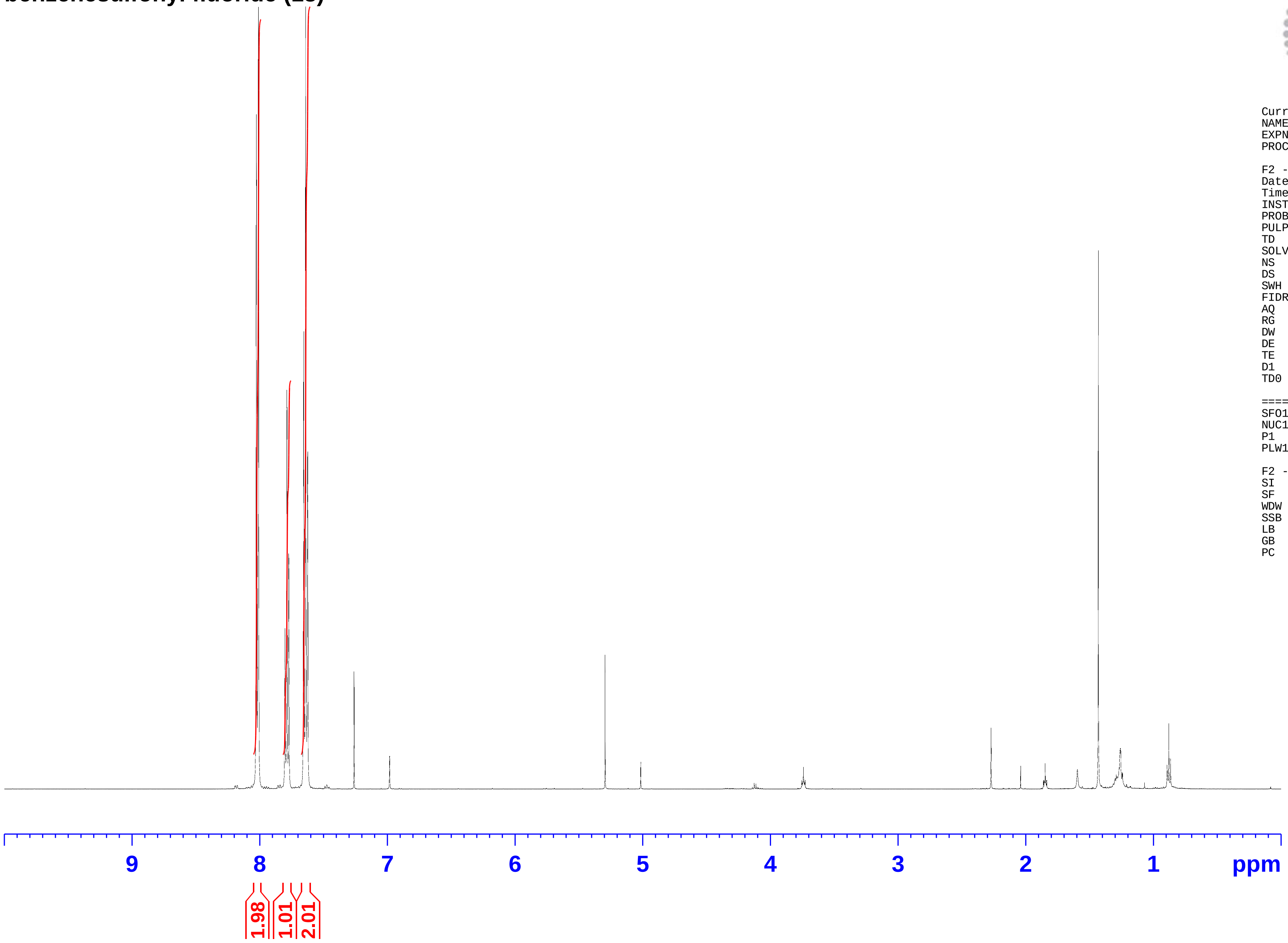


```
Current Data Parameters
NAME
EXPNO                    1
PROCNO                   1

F2 - Acquisition Parameters
Date_             20170518
Time                 16.19
INSTRUM              spect
PROBHD    5 mm QNP 1H/13
PULPROG               zg30
TD                   65536
SOLVENT              CDCl3
NS                      16
DS                       2
SWH          10000.000 Hz
FIDRES        0.152588 Hz
AQ           3.2767999 sec
RG                     161
DW              50.000 usec
DE                6.50 usec
TE               298.2 K
D1          1.00000000 sec
TD0                      1

======== CHANNEL f1 ========
SFO1       500.1330885 MHz
NUC1                    1H
P1               10.00 usec
PLW1       25.00000000 W

F2 - Processing parameters
SI                   65536
SF         500.1300129 MHz
WDW                     EM
SSB      0
LB                0.30 Hz
GB       0
PC                    1.00
```

benzenesulfonyl fluoride (13)

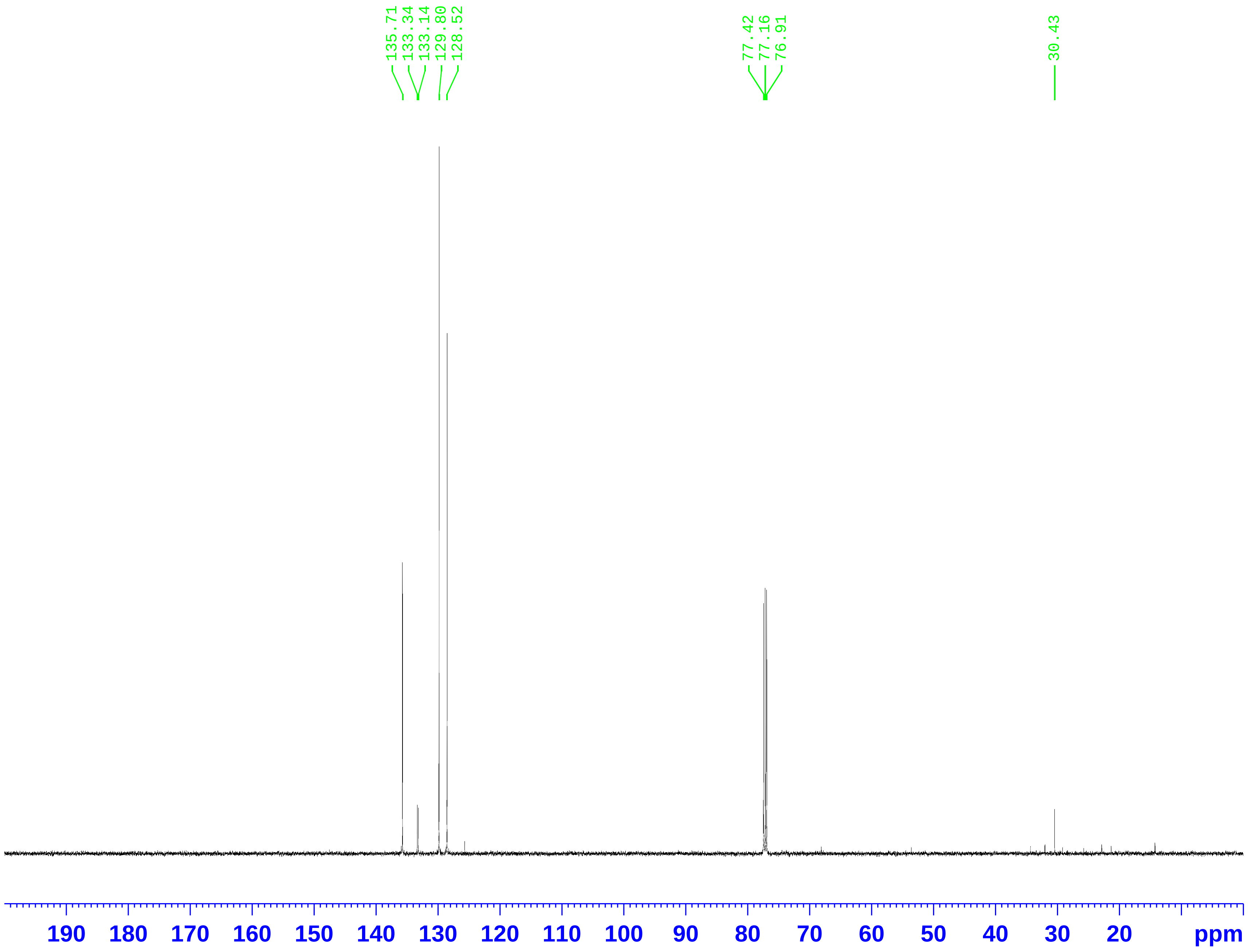


```
Current Data Parameters
NAME
EXPNO                    3
PROCNO                   1

F2 - Acquisition Parameters
Date_             20170519
Time                 14.14
INSTRUM              spect
PROBHD   5 mm QNP 1H/13
PULPROG            zgpg30
TD                   65536
SOLVENT              CDCl3
NS                     256
DS                       4
SWH            29761.904 Hz
FIDRES          0.454131 Hz
AQ             1.1010048 sec
RG                    2050
DW                  16.800 usec
DE                    6.50 usec
TE                   298.2 K
D1            2.00000000 sec
D11           0.03000000 sec
TD0                      1

======== CHANNEL f1 ========
SFO1         125.7703637 MHz
NUC1                   13C
P1                    7.50 usec
PLW1         92.00000000 W

======== CHANNEL f2 ========
SFO2         500.1320005 MHz
NUC2                    1H
CPDPRG[2           waltz16
PCPD2                80.00 usec
PLW2         25.00000000 W
PLW12         0.39063001 W
PLW13         0.19648001 W

F2 - Processing parameters
SI                   32768
SF           125.7577742 MHz
WDW                     EM
SSB      0
LB                    1.00 Hz
GB       0
PC                    1.40
```

methyl N-phenylcarbamate (14)

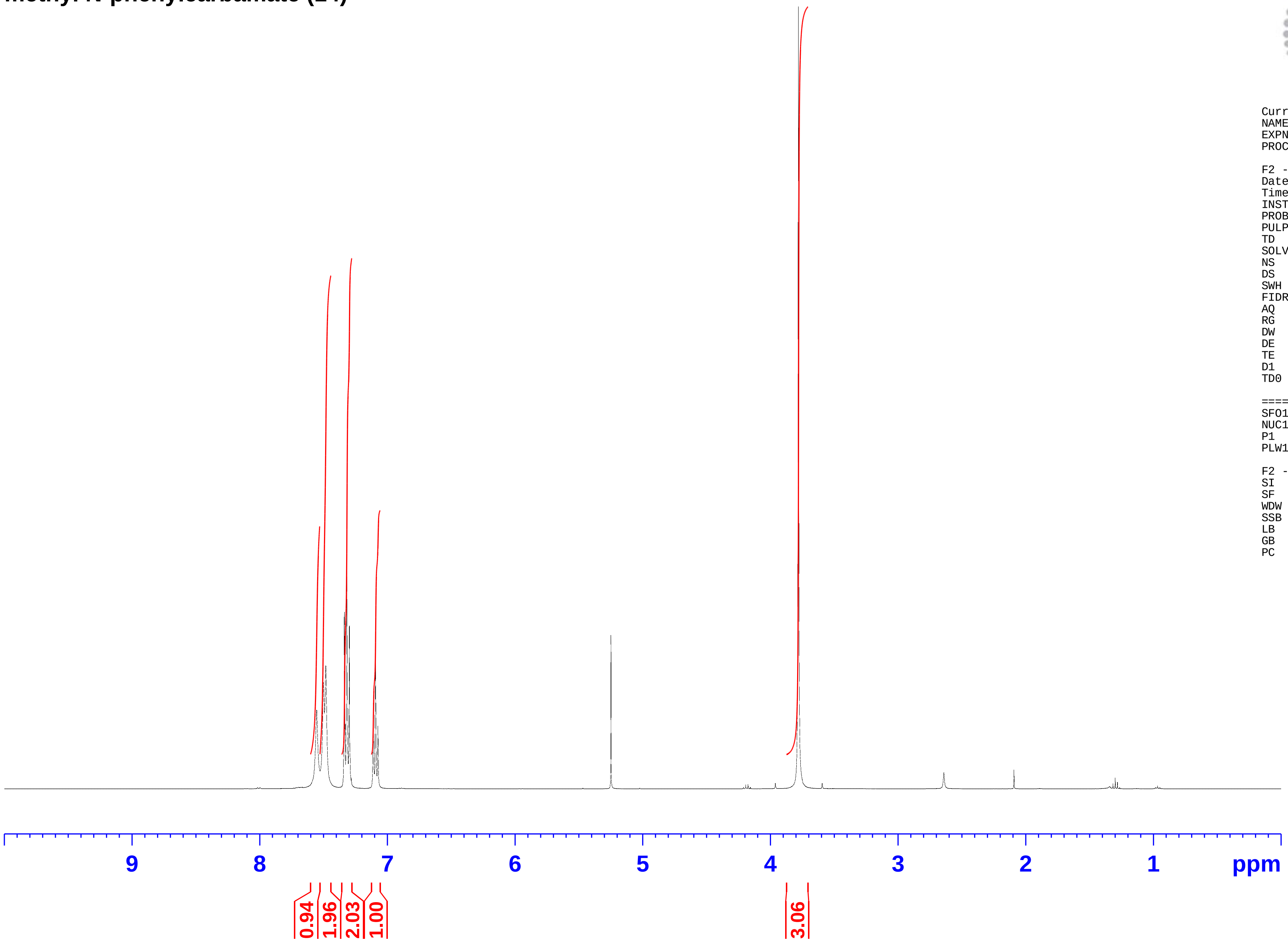


```
Current Data Parameters
NAME
EXPNO                   1
PROCNO                  1

F2 - Acquisition Parameters
Date_            20170530
Time                13.24
INSTRUM             spect
PROBHD   5 mm PADUL 13C
PULPROG              zg30
TD                 131072
SOLVENT             CDCl3
NS                     16
DS                      4
SWH             12019.230 Hz
FIDRES           0.091699 Hz
AQ              5.4525952 sec
RG                  10.29
DW                 41.600 usec
DE                  12.17 usec
TE                  298.2 K
D1             0.10000000 sec
TD0                     1

======== CHANNEL f1 ========
SFO1          400.1324710 MHz
NUC1                   1H
P1                  10.00 usec
PLW1          20.00000000 W

F2 - Processing parameters
SI                 131072
SF            400.1300000 MHz
WDW                    EM
SSB      0
LB                   0.10 Hz
GB       0
PC                   1.00
```

methyl N-phenylcarbamate (14)

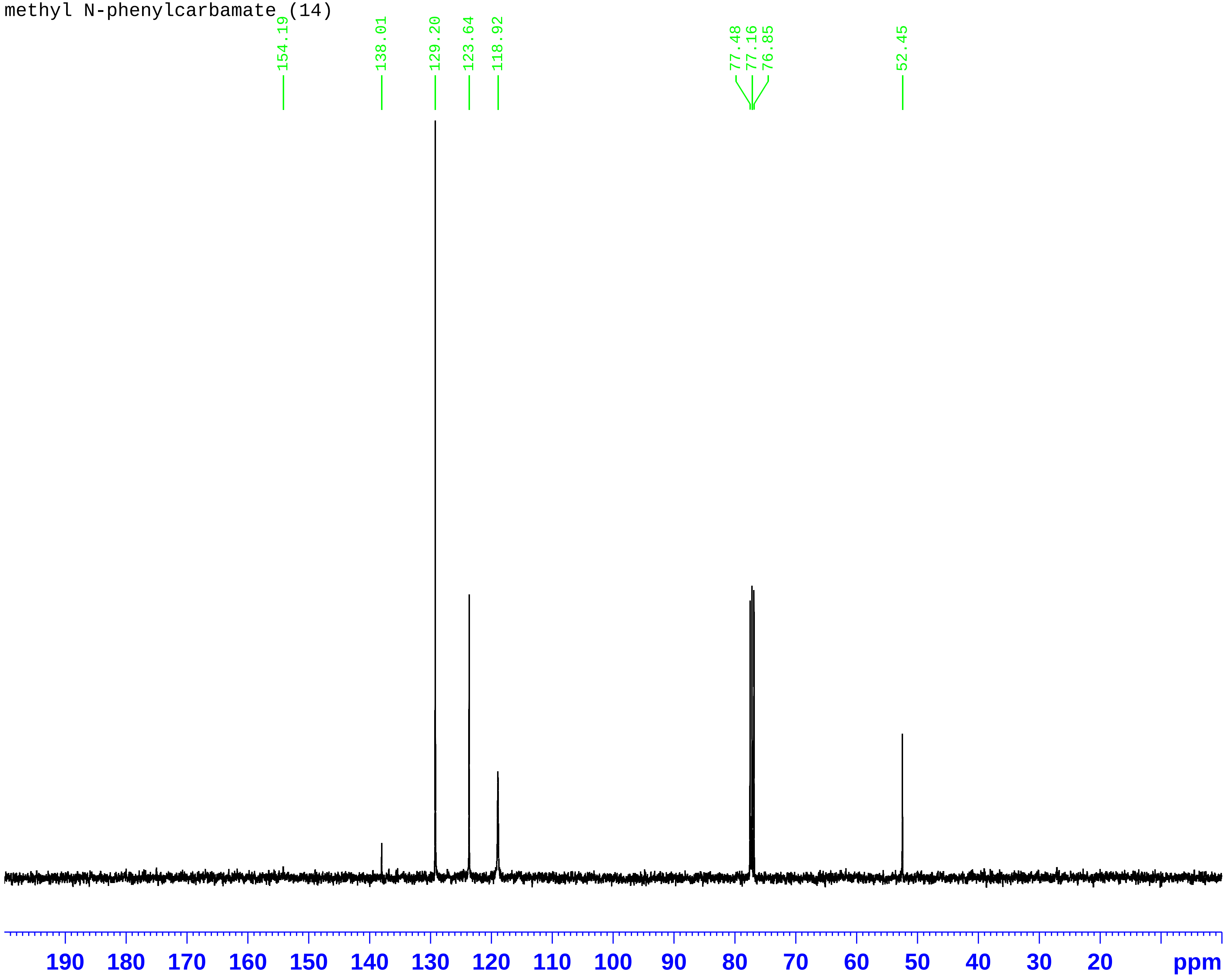


```
Current Data Parameters
NAME
EXPNO                    11
PROCNO                    1

F2 - Acquisition Parameters
Date_              20181112
Time                  19.42
INSTRUM               spect
PROBHD    5 mm PADUL 13C
PULPROG               udeft
TD                    17996
SOLVENT               CDCl3
NS                      256
DS                        0
SWH           25000.000 Hz
FIDRES         1.389198 Hz
AQ            0.3599200 sec
RG                   196.14
DW              20.000 usec
DE                8.66 usec
TE                 303.2 K
D1           3.00000000 sec
D11          0.03000000 sec
D12          0.00002000 sec
D20        200.00000000 sec
TD0                       1

======== CHANNEL f1 ========
SFO1        100.6238346 MHz
NUC1                    13C
P1               10.00 usec
P13            2000.00 usec
P26             500.00 usec
PLW1        36.00000000 W
SPNAM[5]    Crp60comp.4
SPOAL5                0.500
SPOFFS5   0 Hz
SPW5         5.50040007 W
SPNAM[8] Crp60,0.5,20.1
SPOAL8                0.500
SPOFFS8   0 Hz
SPW8         5.50040007 W

======== CHANNEL f2 ========
SFO2        400.1316005 MHz
NUC2                     1H
CPDPRG[2            waltz64
PCPD2            90.00 usec
PLW2        20.00000000 W
PLW12        0.24691001 W

F2 - Processing parameters
SI                   262144
SF          100.6127538 MHz
WDW                      EM
SSB       0
LB                  2.00 Hz
GB        0
PC                     1.40
```

N-phenylethenesulfonamide (15)

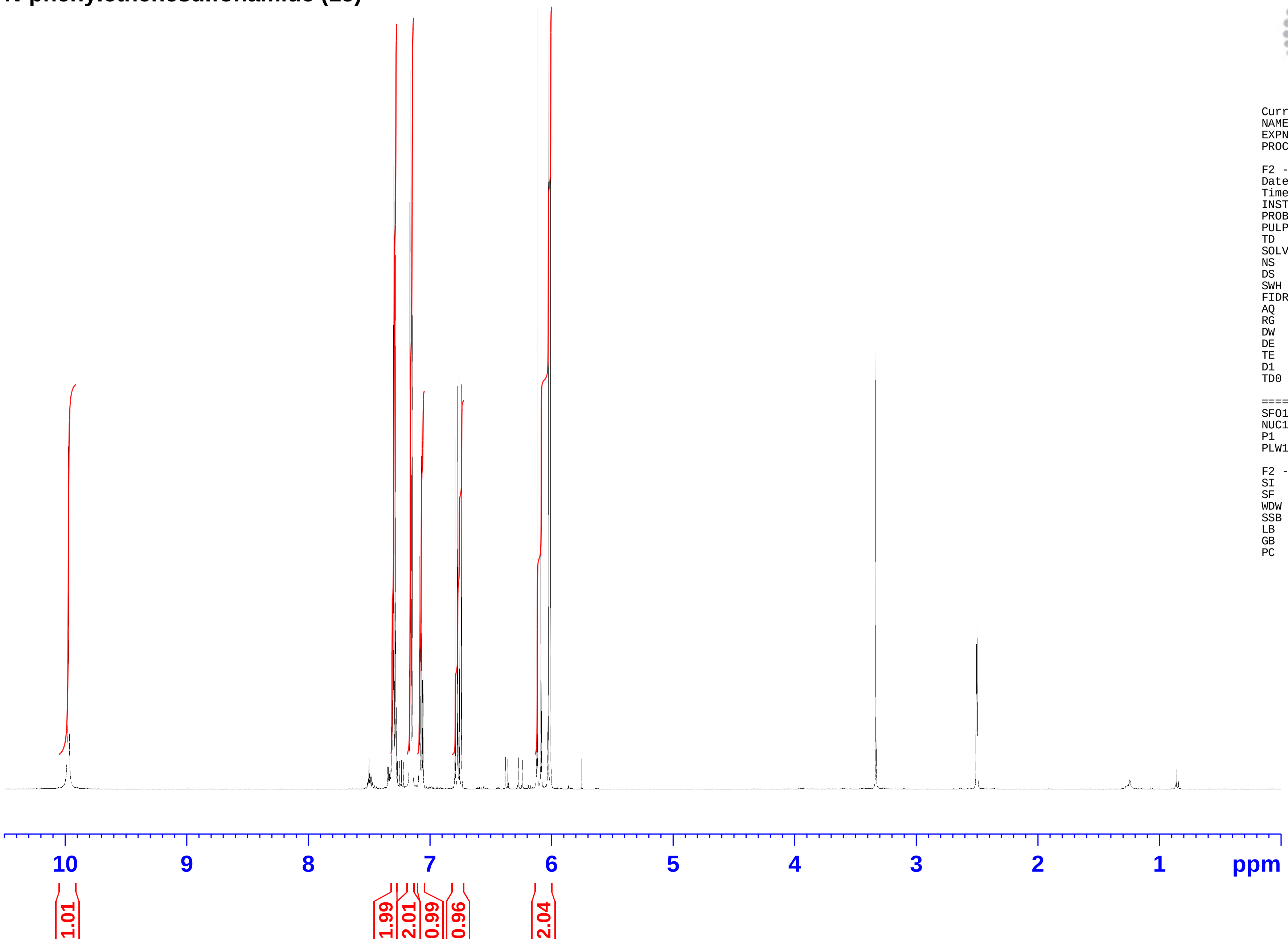

Current Data Parameters	
NAME	
EXPNO	1
PROCNO	1
F2 - Acquisition Parameters	
Date_	20170406
Time	13.48
INSTRUM	spect
PROBHD	5 mm QNP 1H/13
PULPROG	zg30
TD	65536
SOLVENT	DMSO
NS	16
DS	2
SWH	10000.000 Hz
FIDRES	0.152588 Hz
AQ	3.2767999 sec
RG	161
DW	50.000 usec
DE	6.50 usec
TE	298.2 K
D1	1.00000000 sec
TD0	1
======== CHANNEL f1 ========	
SFO1	500.1330885 MHz
NUC1	1H
P1	10.00 usec
PLW1	25.00000000 W
F2 - Processing parameters	
SI	65536
SF	500.1300047 MHz
WDW	EM
SSB	0
LB	0.30 Hz
GB	0
PC	1.00

N-phenylethenesulfonamide (15)

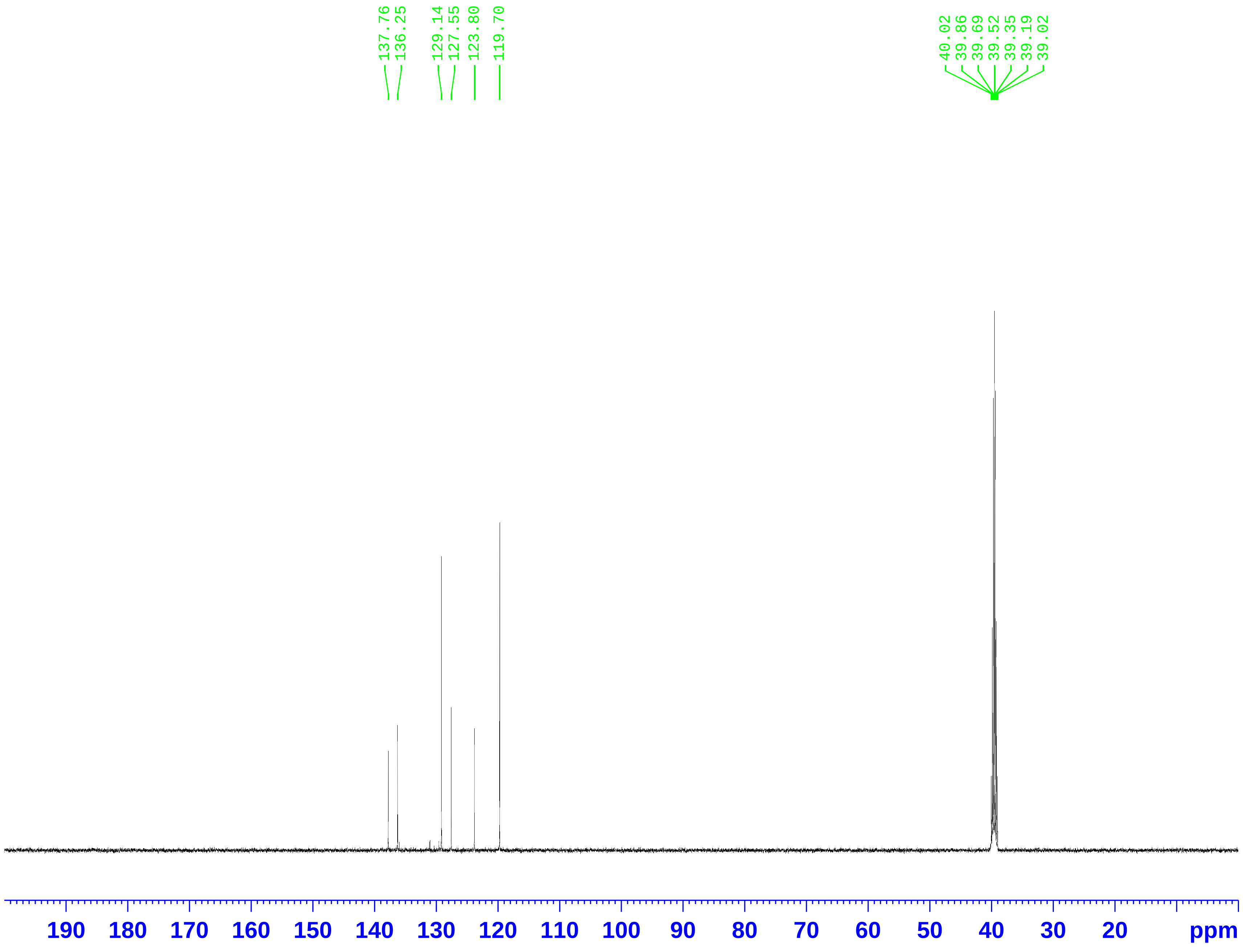

UNIVERSITY OF DUNDEE

```
Current Data Parameters
NAME
EXPNO                    2
PROCNO                   1

F2 - Acquisition Parameters
Date_             20170406
Time                 14.06
INSTRUM              spect
PROBHD    5 mm QNP 1H/13
PULPROG            zgpg30
TD                   65536
SOLVENT               DMSO
NS                     256
DS                       4
SWH             29761.904 Hz
FIDRES           0.454131 Hz
AQ              1.1010048 sec
RG                    2050
DW                  16.800 usec
DE                    6.50 usec
TE                   298.2 K
D1             2.00000000 sec
D11            0.03000000 sec
TD0                      1

======== CHANNEL f1 ========
SFO1          125.7703637 MHz
NUC1                   13C
P1                    7.50 usec
PLW1          92.00000000 W

======== CHANNEL f2 ========
SFO2          500.1320005 MHz
NUC2                    1H
CPDPRG[2           waltz16
PCPD2                80.00 usec
PLW2          25.00000000 W
PLW12          0.39063001 W
PLW13          0.19648001 W

F2 - Processing parameters
SI                   32768
SF            125.7578493 MHz
WDW                     EM
SSB      0
LB                    1.00 Hz
GB       0
PC                    1.40
```

1-phenylprop-2-en-1-ol (18)

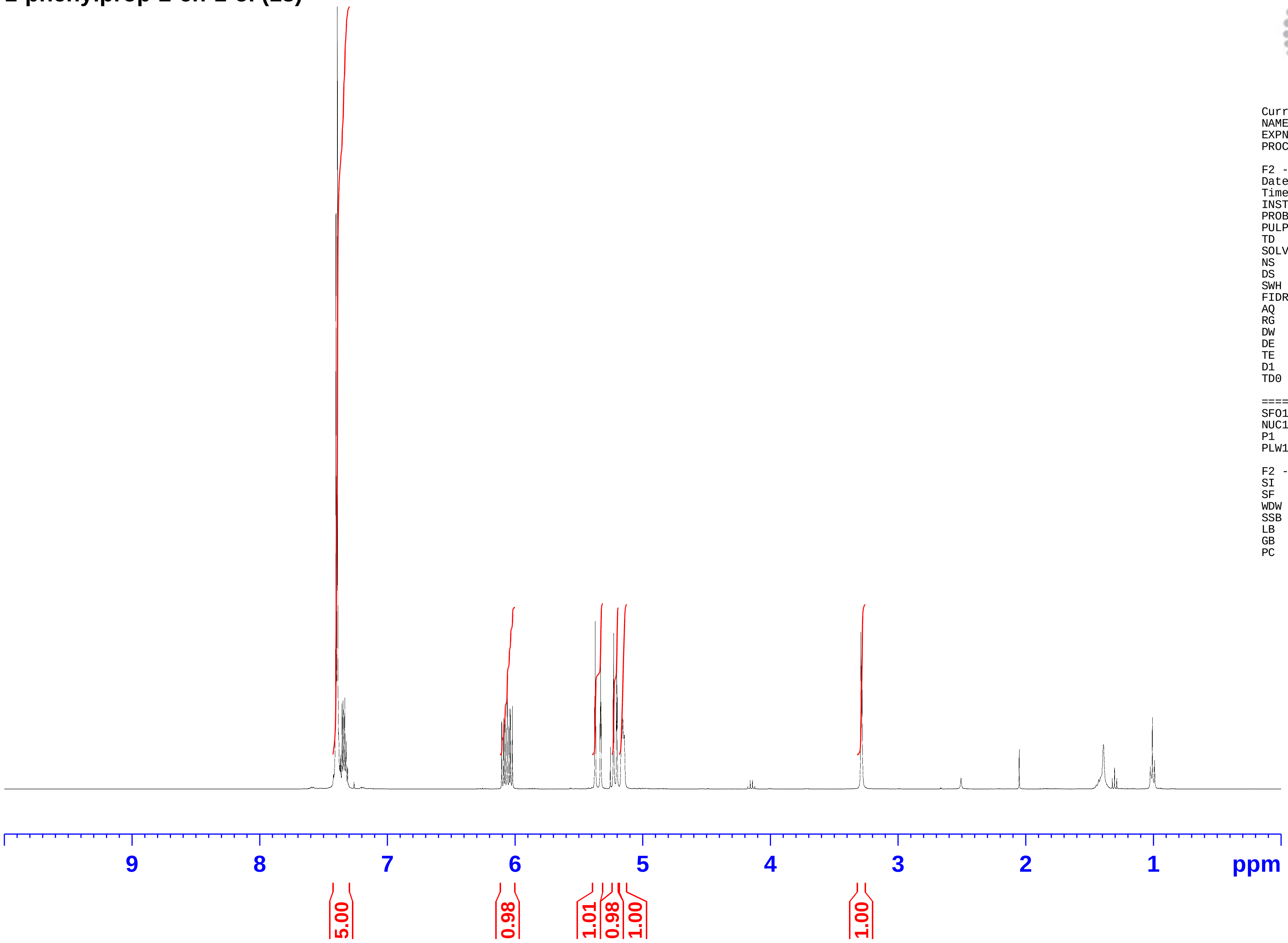


```
Current Data Parameters
NAME
EXPNO                     1
PROCNO                    1

F2 - Acquisition Parameters
Date_              20170524
Time                  16.41
INSTRUM               spect
PROBHD   5 mm PADUL 13C
PULPROG                zg30
TD                   131072
SOLVENT               CDCl3
NS                       16
DS                        4
SWH               12019.230 Hz
FIDRES             0.091699 Hz
AQ                5.4525952 sec
RG                    10.29
DW                   41.600 usec
DE                    12.17 usec
TE                    298.2 K
D1               0.10000000 sec
TD0                       1

======== CHANNEL f1 ========
SFO1            400.1324710 MHz
NUC1                     1H
P1                    10.00 usec
PLW1            20.00000000 W

F2 - Processing parameters
SI                   131072
SF              400.1300091 MHz
WDW                      EM
SSB      0
LB                     0.10 Hz
GB       0
PC                     1.00
```

1-phenylprop-2-en-1-one (16)

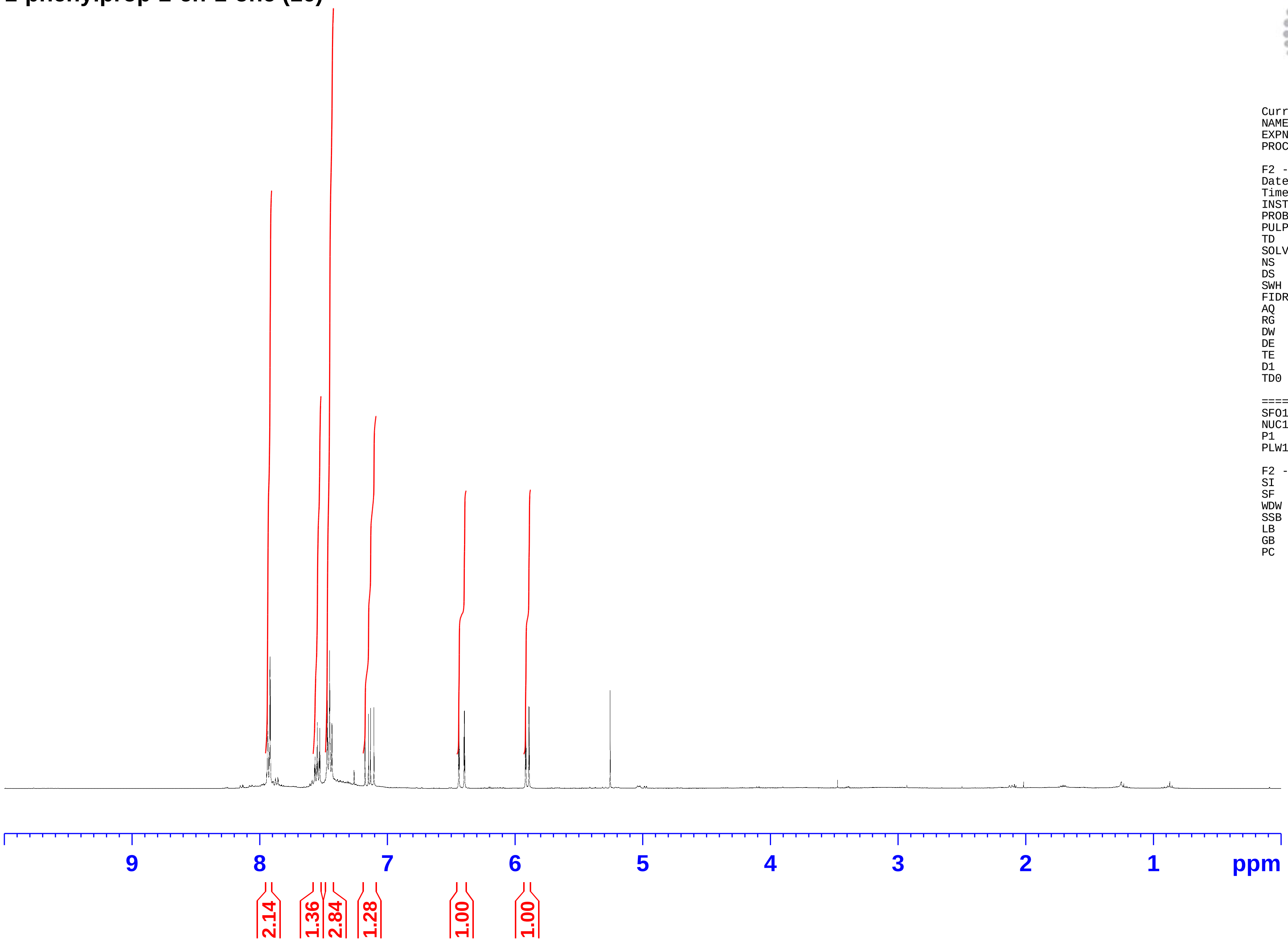

Current Data Parameters	
NAME	
EXPNO	1
PROCNO	1
F2 - Acquisition Parameters	
Date_	20170530
Time	12.59
INSTRUM	spect
PROBHD	5 mm PADUL 13C
PULPROG	zg30
TD	131072
SOLVENT	CDCl3
NS	16
DS	4
SWH	12019.230 Hz
FIDRES	0.091699 Hz
AQ	5.4525952 sec
RG	31.23
DW	41.600 usec
DE	12.17 usec
TE	298.2 K
D1	0.10000000 sec
TD0	1
======== CHANNEL f1 ========	
SFO1	400.1324710 MHz
NUC1	1H
P1	10.00 usec
PLW1	20.00000000 W
F2 - Processing parameters	
SI	131072
SF	400.1300094 MHz
WDW	EM
SSB	0
LB	0.10 Hz
GB	0
PC	1.00

1-phenylprop-2-en-1-one (16)

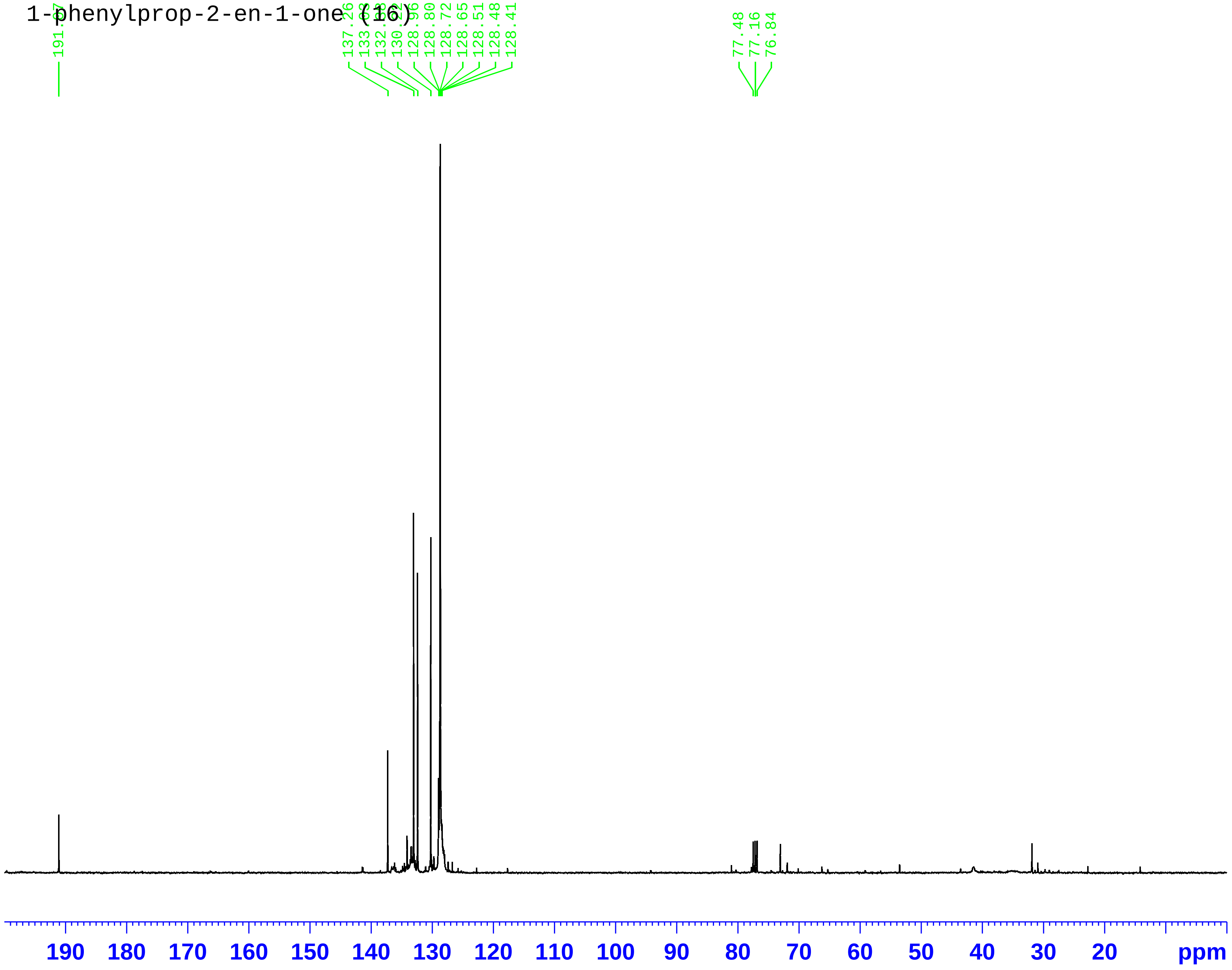


```
Current Data Parameters
NAME
EXPNO                     3
PROCNO                    1

F2 - Acquisition Parameters
Date_              20170530
Time                  15.15
INSTRUM               spect
PROBHD     5 mm PADUL 13C
PULPROG               udeft
TD                    17996
SOLVENT               CDCl3
NS                      256
DS                        0
SWH           25000.000 Hz
FIDRES         1.389198 Hz
AQ            0.3599200 sec
RG                   196.14
DW               20.000 usec
DE                 8.66 usec
TE                  298.2 K
D1           3.00000000 sec
D11          0.03000000 sec
D12          0.00002000 sec
D20        200.00000000 sec
TD0                       1

======== CHANNEL f1 ========
SFO1        100.6238346 MHz
NUC1                    13C
P1                10.00 usec
P13             2000.00 usec
P26              500.00 usec
PLW1         36.00000000 W
SPNAM[5]     Crp60comp.4
SPOAL5                0.500
SPOFFS5   0 Hz
SPW5          5.50040007 W
SPNAM[8] Crp60,0.5,20.1
SPOAL8                0.500
SPOFFS8   0 Hz
SPW8          5.50040007 W

======== CHANNEL f2 ========
SFO2        400.1316005 MHz
NUC2                     1H
CPDPRG[2            waltz64
PCPD2             90.00 usec
PLW2         20.00000000 W
PLW12         0.24691001 W

F2 - Processing parameters
SI                   262144
SF          100.6127678 MHz
WDW                      EM
SSB       0
LB                  2.00 Hz
GB        0
PC                     1.40
```

Boc-Arg-OMe (19)

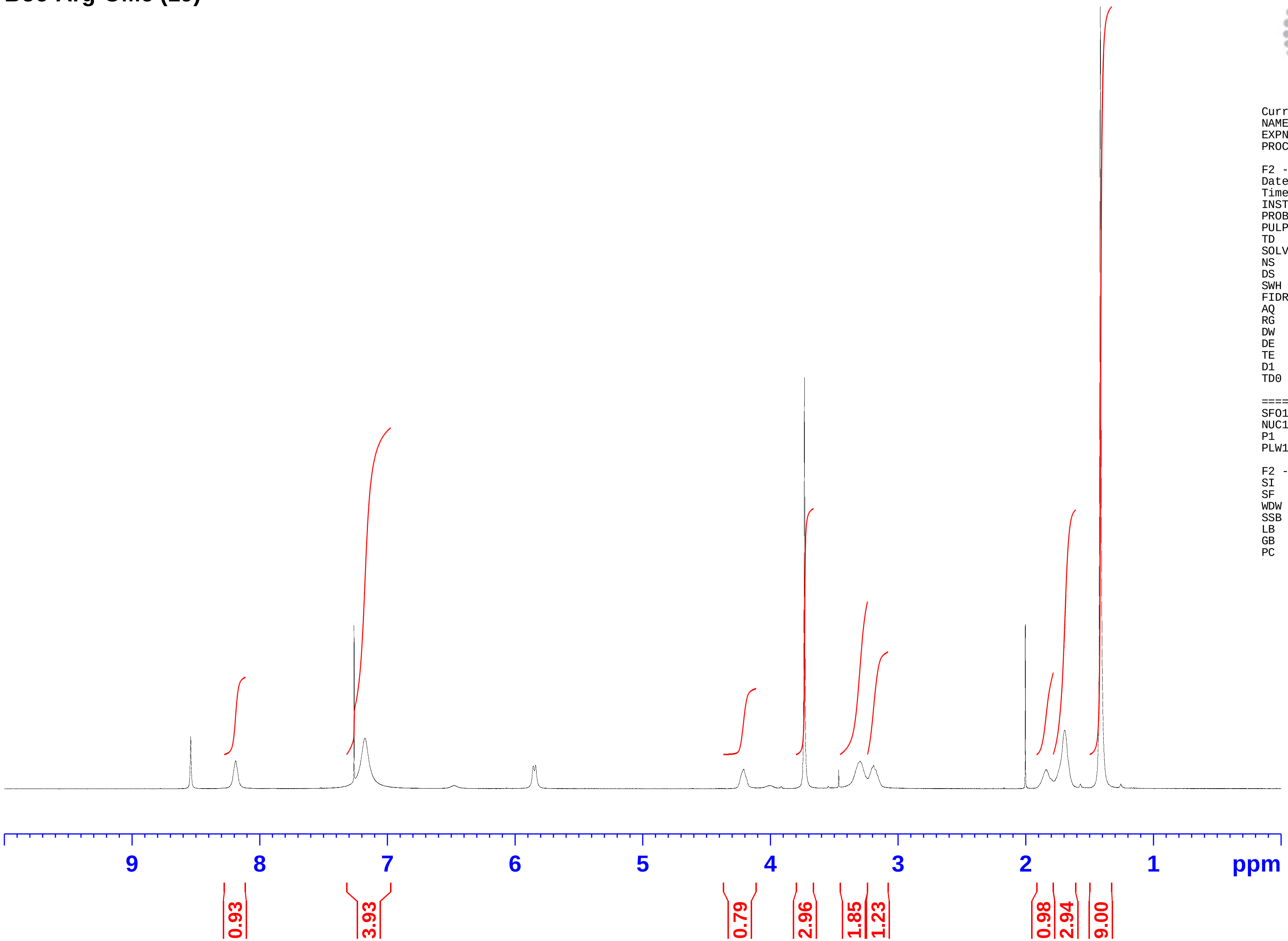


```
Current Data Parameters
NAME
EXPNO                    1
PROCNO                   1

F2 - Acquisition Parameters
Date_             20171103
Time                 11.35
INSTRUM              spect
PROBHD   5 mm PADUL 13C
PULPROG               zg30
TD                  131072
SOLVENT              CDCl3
NS                      16
DS                       4
SWH              12019.230 Hz
FIDRES            0.091699 Hz
AQ               5.4525952 sec
RG                  127.77
DW                  41.600 usec
DE                   12.17 usec
TE                   298.2 K
D1              0.10000000 sec
TD0                      1

======== CHANNEL f1 ========
SFO1           400.1324710 MHz
NUC1                    1H
P1                   10.00 usec
PLW1           20.00000000 W

F2 - Processing parameters
SI                  131072
SF             400.1300096 MHz
WDW                     EM
SSB      0
LB                    0.10 Hz
GB       0
PC                    1.00
```

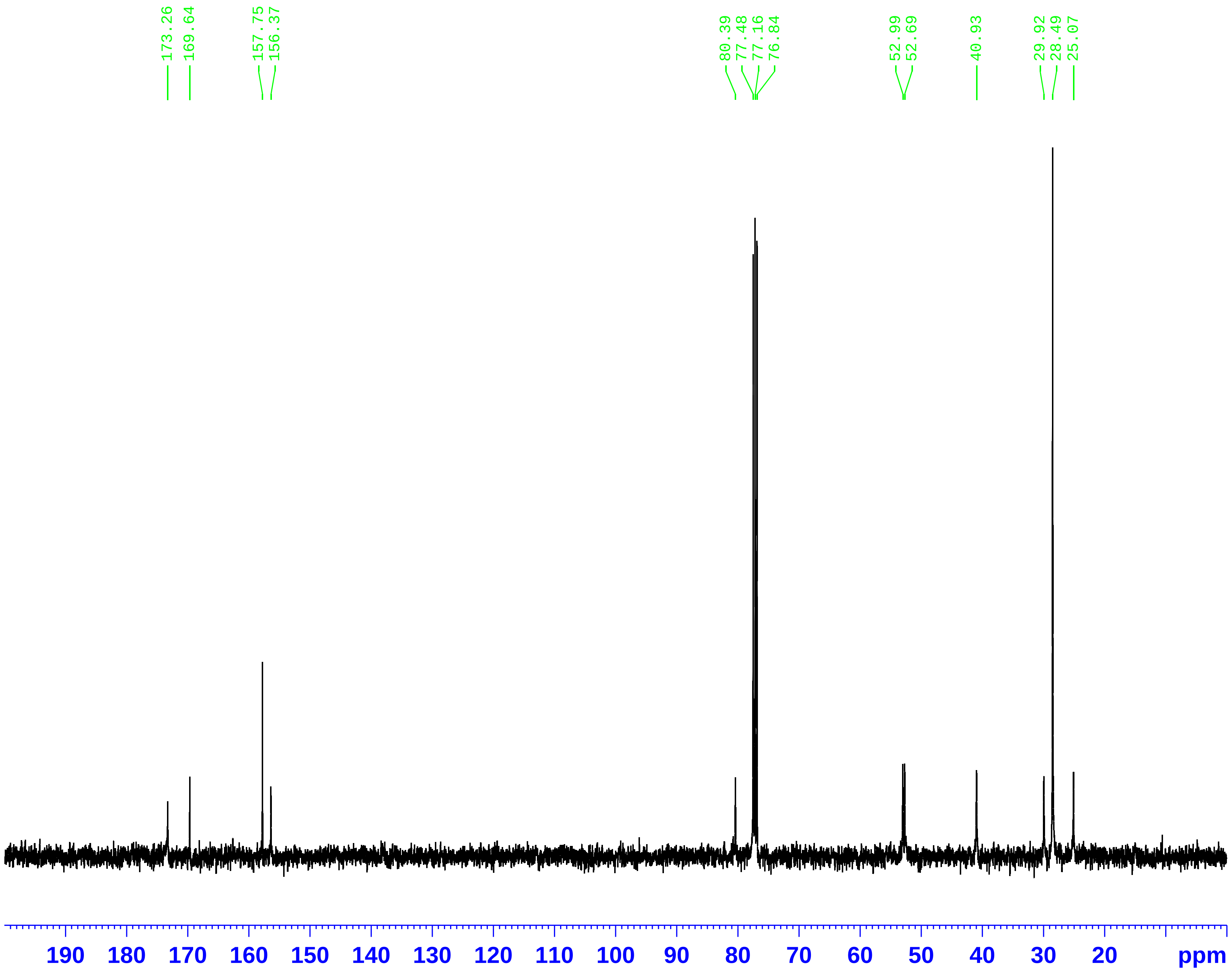

UNIVERSITY OF DUNDEE

```
Current Data Parameters
NAME
EXPNO                     3
PROCNO                    1

F2 - Acquisition Parameters
Date_              20171103
Time                  12.07
INSTRUM               spect
PROBHD    5 mm PADUL 13C
PULPROG               udeft
TD                    17996
SOLVENT               CDCl3
NS                      256
DS                        0
SWH              25000.000 Hz
FIDRES            1.389198 Hz
AQ               0.3599200 sec
RG                   196.14
DW                   20.000 usec
DE                     8.66 usec
TE                    298.2 K
D1               3.00000000 sec
D11              0.03000000 sec
D12              0.00002000 sec
D20            200.00000000 sec
TD0                       1

======== CHANNEL f1 ========
SFO1          100.6238346 MHz
NUC1                      13C
P1                      10.00 usec
P13                   2000.00 usec
P26                    500.00 usec
PLW1              36.00000000 W
SPNAM[5]        Crp60comp.4
SPOAL5                  0.500
SPOFFS5   0 Hz
SPW5               5.50040007 W
SPNAM[8] Crp60,0.5,20.1
SPOAL8                  0.500
SPOFFS8   0 Hz
SPW8               5.50040007 W

======== CHANNEL f2 ========
SFO2          400.1316005 MHz
NUC2                       1H
CPDPRG[2              waltz64
PCPD2                   90.00 usec
PLW2              20.00000000 W
PLW12              0.24691001 W

F2 - Processing parameters
SI                     262144
SF             100.6127558 MHz
WDW                        EM
SSB       0
LB                       2.00 Hz
GB        0
PC                       1.40
```
